# Tumor Microenvironment Profiling Identifies Prognostic Signatures and Suggests Immunotherapeutic Benefits in Neuroblastoma

**DOI:** 10.3389/fcell.2022.814836

**Published:** 2022-04-14

**Authors:** Chenzhao Feng, Ting Li, Jun Xiao, Jing Wang, Xinyao Meng, Huizhong Niu, Bin Jiang, Lei Huang, Xiaogeng Deng, Xueqiang Yan, Dianming Wu, Yifan Fang, Yu Lin, Feng Chen, Xiaojuan Wu, Xiang Zhao, Jiexiong Feng

**Affiliations:** ^1^ Department of Pediatric Surgery, Tongji Hospital, Tongji Medical College, Huazhong University of Science and Technology, Wuhan, China; ^2^ Department of General Surgery, Children’s Hospital of Hebei Province, Shijiazhuang, China; ^3^ Department of General Surgery, Children’s Hospital of Nanjing Medical University, Nanjing, China; ^4^ Department of Pediatric Surgery, Sun Yat-Sen Memorial Hospital, Sun Yat-Sen University, Guangzhou, China; ^5^ Department of Pediatric Surgery, Wuhan Children’s Hospital (Wuhan Maternal and Child Healthcare Hospital), Tongji Medical College, Huazhong University of Science and Technology, Wuhan, China; ^6^ Department of Pediatric Surgery, Fujian Provincial Maternity and Children’s Hospital, Fuzhou, China; ^7^ Department of Pediatric Surgery, Fujian Medical University Union Hospital, Fuzhou, China

**Keywords:** neuroblastoma, tumor microenvironment, immunotherapy, pan-cancer, pediatric cancer

## Abstract

The tumor microenvironment (TME) influences disease initiation and progression. Cross-talks of cells within TME can affect the efficacy of immunotherapies. However, a precise, concise, and comprehensive TME landscape in neuroblastoma (NB) has not been established. Here, we profiled the TME landscape of 498 NB-related patients on a self-curated gene list and identified three prognostic TMEsubgroups. The differentially expressed genes in these three TMEsubgroups were used to construct a genetic signature of the TME landscape and characterize three GeneSubgroups. The subgroup with the worst overall survival prognosis, the TMEsubgroup/GeneSubgroup3, lacked immune cell infiltration and received the highest scores of *MYCN*- and *ALK*-related signatures and lowest scores of immune pathways. Additionally, we found that the GeneSubgroup3 might be benefited from anti-GD2 instead of anti-PD-1 therapy. We further created a 48-gene signature, the TMEscore, to infer prognosis and validated it in three independent NB cohorts and a pan-cancer cohort of 9,460 patients. We did RNA-seq on 16 samples and verified that TMEscore was higher in patients with stage 3/4 than stage 1/2 diseases. The TMEscore could also predict responses for several immunotherapies. After adding clinical features, we found that the nomogram-based score system, the TMEIndex, surpassed the current risk system at predicting survivals. Our analysis explained TME at the transcriptome level and paved the way for immunotherapies in NB.

## Introduction

Neuroblastoma (NB), derived from the sympathetic nervous system with a broad spectrum of clinical manifestations, is the most common extracranial solid tumor in childhood (Jones et al., 2019). Several genetic alterations, such as *MYCN*-amplification, *ALK* mutation, and segmental chromosomal alterations, have been found to be associated with the oncogenesis and progression of NB. Despite surgeries, chemotherapies, and immunotherapies having been administered to patients, the event-free survivals of high-risk phenotypes remain low (Park and Cheung, 2020). Thus, new drug targets and synergistic combination therapies are needed to be unearthed.

Cellular bidirectional communications are vital for tumor cells growth, and the tumor microenvironment (TME) influences disease initiation, progression, and patient prognosis (Galon and Bruni, 2020). A subset of tumor-associated macrophages within the TME might contribute to disease progression despite immunosurveillance, but other populations might support the efficacy of anticancer therapies (Vitale et al., 2019). Myeloid-derived suppressor cells (MDSCs) and regulatory T cells (Treg cells) infiltrate the TME to disrupt the immune surveillance and inhibit cytolytic activities (Ferreira et al., 2019; Veglia et al., 2021). In NB, tumor-associated macrophages produce IL-1β and tumor necrosis factor-α to regulate arginine metabolism and thus promote cell proliferation (Fultang et al., 2019). NB tumor cells show a low tumor mutational burden and escape the immune system by downregulating HLA (Ferreira et al., 2019; Veglia et al., 2021). These results suggest that investigations of TME in NB could reveal the potential mechanisms of tumor progression.

Immune-checkpoint therapies have been demonstrated to be effective in a number of cancers (Horn et al., 2018; André et al., 2020; Gutzmer et al., 2020). High-risk NB patients lack tumor-infiltrating lymphocytes and programmed-death ligand-1 (PD-L1) expression, indicating that these patients are not qualified for anti-PD-L1 regimes (Srinivasan et al., 2018). Instead, a disialoganglioside GD2 is presented at high density on all tumors, and anti-GD2 therapies are impressive in improving the survivals of NB patients (Ladenstein et al., 2018; Heczey et al., 2020). However, it is not determined which patients would benefit from diverse immunotherapies in a transcriptome aspect.

In this study, we identified NB-specific TME markers from single-cell analysis and profiled the TME landscape and its genetic background from 498 NB patients. Among three subgroups, the one with the worst overall survival prognosis, TMEsubgroup3, lacked immune cell infiltration. At the same time, the corresponding GeneSubgroup3 received the highest scores of *MYCN*- and *ALK*-related signatures and lowest scores of immune pathways but would benefit from anti-GD2 therapies. We further created a 48-gene signature, the TMEscore, to infer prognosis and validated its accuracy in three independent NB cohorts and a pan-cancer cohort of 9,460 patients. We verified that TMEscore was higher in stage 3/4 than stage 1/2 patients by RNA-seq on 16 samples. After combining with clinical information, we found that the nomogram-based score system, the TMEIndex, surpassed the current Children's Oncology Group (COG) risk classification system at predicting survivals. Overall, our analysis explained TME phenotype at a transcriptome level and paved the way for precise immunotherapies for NB.

## Materials and Methods

### Neuroblastoma Bulk RNA-Seq Data Acquisition

In this study, we gathered three microarray datasets and one RNA-seq dataset, GSE49710 (SEQC), E-MTAB-8248, and E-MTAB-179, all based on Agilent-020382 Human Custom Microarray 44 k, with 498, 478, and 223 patients, respectively, for NB analysis. These datasets were downloaded from Gene Expression Omnibus (GEO, https://www.ncbi.nlm.nih.gov/geo/) and ArrayExpress (https://www.ebi.ac.uk/arrayexpress/). The raw files were preprocessed using the RMA algorithm for background adjustment and quantile normalization by the *limma* functions “backgroundCorrect” and “normalizeBetweenArrays” (Ritchie et al., 2015). Probes were annotated into gene symbols by GPL16876. When encountered with distinct probes corresponding to the same genes, probes with the maximum median values were accepted. Level 4 gene-expression data of Therapeutically Applicable Research To Generate Effective Treatments (TARGET) were downloaded from the UCSC Xena browser (GDC hub: https://gdc.xenahubs.net). For the TARGET dataset, RNA-sequencing data [fragments per kilobase of exon per million mapped fragments (FPKM) values] were transformed into transcripts per kilobase million (TPM) values. The Ensemble IDs were annotated into gene symbols, and protein-coding genes were extracted using *gencode. v22. gtf* (https://www.gencodegenes.org/) according to The Cancer Genome Atlas (TCGA) pipeline (https://docs.gdc.cancer.gov/Data/Bioinformatics_Pipelines/Expression_mRNA_Pipeline/)*.*

To address the batch effect between each chip and other sequencing backgrounds, we utilized the “combat” function in *sva* R package (http://bioconductor.org/packages/release/bioc/html/sva.html). The batch-corrected expression matrix was used for downstream analysis.

Clinical information, including age, sex, MYCN status, International Neuroblastoma Staging System (INSS) stage, COG risk, overall survival, event-free survival, and their survival time, was retrieved from supplementary files of these studies and is summarized in Supplementary Table S1. We used the SEQC cohort to portray the TME in NB, and the remaining three cohorts were used to validate prognostic signatures.

### Gene Set Construction

Single-cell RNA-seq count data of six NB patients with Chromium Single Cell 30 V3 Reagent Kit were downloaded from GEO (GSE137804) for the NB-specific marker identification. Cell types were annotated by Dong et al. (2020) and retrieved in the supplementary file.

We designed a pipeline to construct a compendium of microenvironment genes related to NB-specific cell subsets. We modified the gene list acquired from Charoentong et al. (2017), deleting markers that expressed on NB tumor cells and adding markers of endothelial cells, fibroblasts, and Schwann cells. Markers were identified in the single-cell RNA-seq data.

First, single-cell RNA-seq counts were preprocessed and visualized by the *Seurat* functions “NormalizeData,” “FindVariableFeatures,” “ScaleData,” “RunPCA,” “FindNeighbors,” “FindClusters,” “RunUMAP,” and “DimPlot” with default parameters (Stuart et al., 2019). Cells were removed when two diverse cell types were grouped into the same cluster. Cell type markers were identified by the “FindAllMarkers” function. The top 20 markers for endothelial cells, fibroblasts, and Schwann cells were added to the gene list directly. Finally, we removed markers that expressed on NB tumor cells in the single-cell data from the list. These procedures gave an NB-peculiar microenvironment backdrop that was composed of 31 types of cells.

### Inference of the Abundances of Cells in Tumor Microenvironment

To calculate the relative proportions of each cell in the biopsy, single-sample gene set enrichment analysis (ssGSEA) was applied to the bulk RNA-seq data of the SEQC cohort. This was achieved by the *GSVA* function “gsva.” We also used the TIMER, CIBERSORT, quanTIseq, MCP-counter, and EPIC algorithms to infer cell infiltrations on the website (http://timer.cistrome.org/).

### Consensus Clustering of Samples

For a given cells × samples matrix, samples were grouped by the unsupervised clustering *K-means* methods. The best number of clusters was determined by the consensus clustering algorithm *via* the *ConsensusClusterPlus* R package.

### Differentially Expressed Genes Analysis

To identify genes contributing to the unbalanced TME and survival outcomes, we did the differentially expressed genes (DEG) analysis across three subgroups identified in Section 2.4 in the SEQC cohort. DEGs were discovered by the *limma* package, with the absolute values of log2(fold-change) greater than 1.4 and an adjusted *p*-value below 0.05. For the DEGs × samples matrix, samples were grouped by the unsupervised clustering *K-means* methods to obtain clusters in transcriptome backgrounds.

### Function and Pathway Analysis

To identify the biological functions of these DEGs in different subgroups, we conducted gene ontology (GO) enrichment analysis by *clusterProfiler* R package, with the *p*-value adjusted by Benjamini–Hochberg methods.

The immune, stromal, and oncological pathways, including CD8 T-effector, antigen processing machinery, immune-checkpoint, epithelial–mesenchymal transition (EMT) markers, pan-fibroblast TGF-β response signature, DNA replication-dependent histones, mismatch repair, nucleotide excision repair, and DNA damage repair, were curated by Zeng et al. (2019). Additionally, we retrieved a 157 *MYCN*-associated gene signature from Valentijn et al. (2012) and 9 telomere maintenance mechanism-associated signatures from Nersisyan et al. (2019).

For the pathway analysis, the principal component analysis was performed on the genes × samples matrix, where genes were from each pathway signature. Then, the principal component 1 of each sample was extracted to serve as the pathway score. This approach has the advantage of focusing the score on the set with the largest block of well-correlated (or anticorrelated) genes in the set while down-weighting contributions from genes that do not interact with other set members.

### Gene Traits Analysis

To explore gene patterns among subgroups, we collected curated cytokines from Immport Cytokine Registry (https://www.immport.org/resources/cytokineRegistry), immune-checkpoint genes from a database (https://www.rndsystems.com/cn/research-area/co–stimulatory-and-co–inhibitory-molecules), and GD2-related synthases from one literature (Sorokin et al., 2020). We also calculated IMPRES (Auslander et al., 2018), a predictor of various immunotherapies responses, and a GD2 positive predictor as the sum of ST8SIA5 and B4GALNT1 (Sorokin et al., 2020).

### Establishment of the TMEscore

To construct a prognostic model predicting NB survivals, we first conducted univariable Cox regressions on all DEGs on the SEQC cohort. Genes that owned hazard ratios (HR) < 1 and *p* < 0.05 were designated as group A and those with HR > 1 and *p* < 0.05 were designated as group B. For group A and group B genes, we conducted principal component analysis on each cohort and extracted principal component 1 as ScoreA and ScoreB, respectively.

$$ScoreA=\;PC1_{genes\;\in \;A\;}$$

$$ScoreB=\;PC1_{genes\;\in \;B}$$

For the *j*th patient, the TMEscore was calculated as:

$$TMEscore_{j}=2\text{∗}ScoreB_{j}-ScoreA_{j}$$

Patients were split in to high- and low- TMEscore groups based on their scores. For each cohort, the best cutoff was determined by the “surv_cutpoint” function in the *survminer* package (https://cran.r-project.org/web/packages/survminer/index.html).

### Evaluation of Prognostic Values of TMEscore in Other Cancers

We downloaded FPKM-normalized RNA-seq data from TCGA pan-cancer projects on the UCSC Xena website (http://xena.ucsc.edu/). This included 9,460 patients with adequate survival information (survival time > 30 days) from 30 cancer types. FPKM values were transformed into TPM values for analysis.

To extend our TMEscore derived from NB, TMEscores were calculated in 30 cancer types (TCGA pan-cancer project) using the same formula earlier, and patients were grouped into high- and low-TMEscore groups. The cutoff values were determined by the “surv_cutpoint” function within each cancer type.

### Evaluation of Predictive Values of TMEscore in Immunotherapies

Five RNA-seq data of different immunotherapy trials and experiments were retrieved and preprocessed under the following pipeline. For the metastatic melanoma with the anti-MAGE-3 cohort (GSE35640, *N* = 55) and the mouse model with the anti-CTLA-4 cohort (GSE63557, *N* = 20), normalized expressions were downloaded. For the urothelial carcinoma with anti-PD-L1 cohort (IMvigor, *N* = 298), counts were accessed with the “IMvigor210CoreBiologies” R package and normalized by the size factors provided by the package. For the melanoma with the anti-PD-1 cohort (GSE78220, *N* = 28), FPKM values were downloaded. For the melanoma cohort with various immunotherapies [TCGA—skin cutaneous melanoma (SKCM), *N* = 36], FPKM values were downloaded and transformed into TPM values. Associated clinical information was retrieved from supplementary files of these studies.

To test whether our TMEscore derived from NB could predict immunotherapy responses, TMEscores were calculated in each cohort mentioned earlier using the same formula, and patients were grouped into high- and low-TMEscore groups. The cutoff values were calculated within each cancer type.

### Establishment of the Nomogram and TMEIndex

To assure the TMEscore was independent of clinical variables, we did uni- and multivariable Cox regression analysis on the TMEscore, age, MYCN status, sex, stage, and COG risk. The nomogram, which could help clinicians make decisions, was established by multivariable Cox regression and visualized by the *rms* R package (https://cran.r-project.org/web/packages/rms/index.html). The total points a patient received in the nomogram were assigned as the TMEIndex. Patients were also split into high- and low- TMEIndex groups by the best cutoff determined by the “surv_cutpoint” function in the *survminer* package.

To compare the prognostic efficacy of the TMEscore and the TMEIndex, we graphed the restricted mean survival curves. We also compared the TMEscore, the TMEIndex, the COG Risk, and the TISscore in the receiver operating characteristic (ROC) curves. The TISscore was calculated as an average value of log^2-^ scale normalized expression of the 18 signature genes (Ayers et al., 2017).

### Patient Samples Collection

We retrospectively collected 16 biopsies of NB by surgeries in six hospitals from January 1, 2019, to March 1, 2021. All the tissue samples included in this study were obtained with approval from the independent Ethics Committee Institutional Review Board of each hospital, and the patients provided written informed consents. Clinical information was obtained through electronic health records.

### RNA Quantification and Qualification

RNA degradation and contamination were monitored on 1% agarose gels. RNA purity was checked using the NanoPhotometer spectrophotometer (IMPLEN, CA, United States). RNA concentration was measured using Qubit RNA Assay Kit in Qubit 2.0 Flurometer (Life Technologies, CA, United States). RNA integrity was assessed using the RNA Nano 6000 Assay Kit of the Bioanalyzer 2,100 system (Agilent Technologies, CA, United States).

### Library Preparation for Transcriptome Sequencing

A total amount of 3 µg RNA per sample was used as input material for the RNA sample preparations. Sequencing libraries were generated using NEBNext Ultra RNA Library Prep Kit for Illumina (NEB, United States) following manufacturer's recommendations, and index codes were added to attribute sequences to each sample. Briefly, messenger RNA was purified from total RNA using poly-T oligo-attached magnetic beads. Fragmentation was carried out using divalent cations under elevated temperature in NEBNext first-strand synthesis reaction buffer (5×). First-strand complementary DNA (cDNA) was synthesized using random hexamer primer and M-MuLV reverse transcriptase (RNase H-). Second-strand cDNA synthesis was subsequently performed using DNA polymerase I and RNase H. Remaining overhangs were converted into blunt ends *via* exonuclease/polymerase activities. After adenylation of 3′ ends of DNA fragments, NEBNext adaptor with hairpin loop structure was ligated to prepare for hybridization. To select cDNA fragments preferentially 150–200 bp in length, the library fragments were purified with the AMPure XP system (Beckman Coulter, Beverly, United States). Then, 3-µl USER enzyme (NEB) was used with size-selected, adaptor-ligated cDNA at 37°C for 15 min followed by 5 min at 95°C before polymerase chain reaction (PCR). Then, PCR was performed with Phusion High-Fidelity DNA polymerase, Universal PCR primers, and Index (X) Primer. At last, PCR products were purified (AMPure XP system), and library quality was assessed on the Agilent Bioanalyzer 2,100 system.

### Clustering and Sequencing

The clustering of the index-coded samples was performed on a cBot Cluster Generation System using TruSeq PE Cluster Kit v3-cBot-HS (Illumia) according to the manufacturer's instructions. After cluster generation, the library preparations were sequenced on an Illumina NovaSeq6000 platform, and 150-bp paired-end reads were generated.

### Quality Control and Quantification of Gene Expression

Raw data (raw reads) of fastq format were firstly processed through in-house Perl scripts. In this step, clean data (clean reads) were obtained by removing reads containing adapter and reads containing ploy-N and low-quality reads from raw data. At the same time, Q20, Q30, and GC content of the clean data were calculated. All the downstream analyses were based on clean data with high quality.

Clean fastq files were then mapped against human reference genome CRh38 using STAR (v2.9.6a) two-pass mode. HTSeq-count was used to count the reads numbers mapped to each gene with the annotation file *gencode. v38. annotation.gtf*. Raw counts of gene expressions were transformed into TPM values for further analysis.

### Immunohistochemistry

One NB tissue chip (N264001, Bioaitech, China) were utilized for CD3 and CD8 immunostaining in accordance with the Specimens in Tissue Chips Collection and use guideline approved by the Ethics Committee of People's Hospital of Xutong County, Henan Province, and subsequent approval by the Ethical Management Committee of Tongji Hospital—Tongji Medical College. Tissue sections were subjected to immunohistochemical (IHC) analysis using the AvidinBiotin Complex (ABC) Vectastain Kit (SP-9001, ZsgbBio) according to the manufacturer's protocol. Antihuman CD3 (A19017, ABclonal) and CD8 (A0663, ABclonal) were used as the primary antibodies. Furthermore, immunostaining was evaluated independently by two pathologists who were blinded to all clinical information.

To quantify CD3^+^ and CD8^+^ cell proportions, first, we randomly selected three 20× regions per sample. This left a total of 12 regions for stage 1 and stage 4 groups, respectively. Next, QuPath (v0.3.2, https://qupath.github.io/) was used to identify total cell numbers and positive cell numbers by default parameters. Positive cell proportions were calculated as positive cell numbers/total cell numbers by QuPath.

### Statistical Analysis

For categorical and continuous data with normal distribution, we applied Chi-square tests and Student t-tests to distinguish the differences between groups. When continuous data were not normally distributed, Wilcoxon rank-sum tests and Kruskal–Wallis rank-sum tests were utilized for two-group and three-group comparisons, respectively. The Pearson correlation test was used to find linear connections between two groups. A *p*-value < 0.05 was considered statistically significant except for emphasis. To account for multiple testing, the *p*-values were adjusted using the Benjamini–Hochberg correction. The heatmaps were plotted by the *ComplexHeatmap* R package (Gu et al., 2016). Kaplan–Meier plots and log-rank tests were used to examine survival differences between subgroups. Time-dependent ROC curves were graphed by the *timeROC* R package (https://cran.r-project.org/web/packages/timeROC/index.html). The forest plots and the alluvial plots were visualized by the *forestplot* (https://cran.r-project.org/web/packages/forestplot/index.html) and the *ggalluvial* (https://cran.r-project.org/web/packages/ggalluvial/index.html) R package. All statistical analyses were two-tailed and done by R (R Foundation, version 3.7.0).

### Ethics Statement

All patients’ samples were obtained according to the Declaration of Helsinki, and each patient signed written informed consent for all the procedures. These procedures were approved by the independent Ethics Committee Institutional Review Board of each hospital.

### Data Deposition and Materials Sharing

All public datasets could be accessed *via* GEO (https://www.ncbi.nlm.nih.gov/geo/) and ArrayExpress (https://www.ebi.ac.uk/arrayexpress/). Raw fastq files of 16 NB samples in the Tongji cohort were deposited on the GEO: GSE182586.

## Results

### Single-Cell Analysis Identified Neuroblastoma-Specific Tumor Microenvironment Markers

**FIGURE 1:**
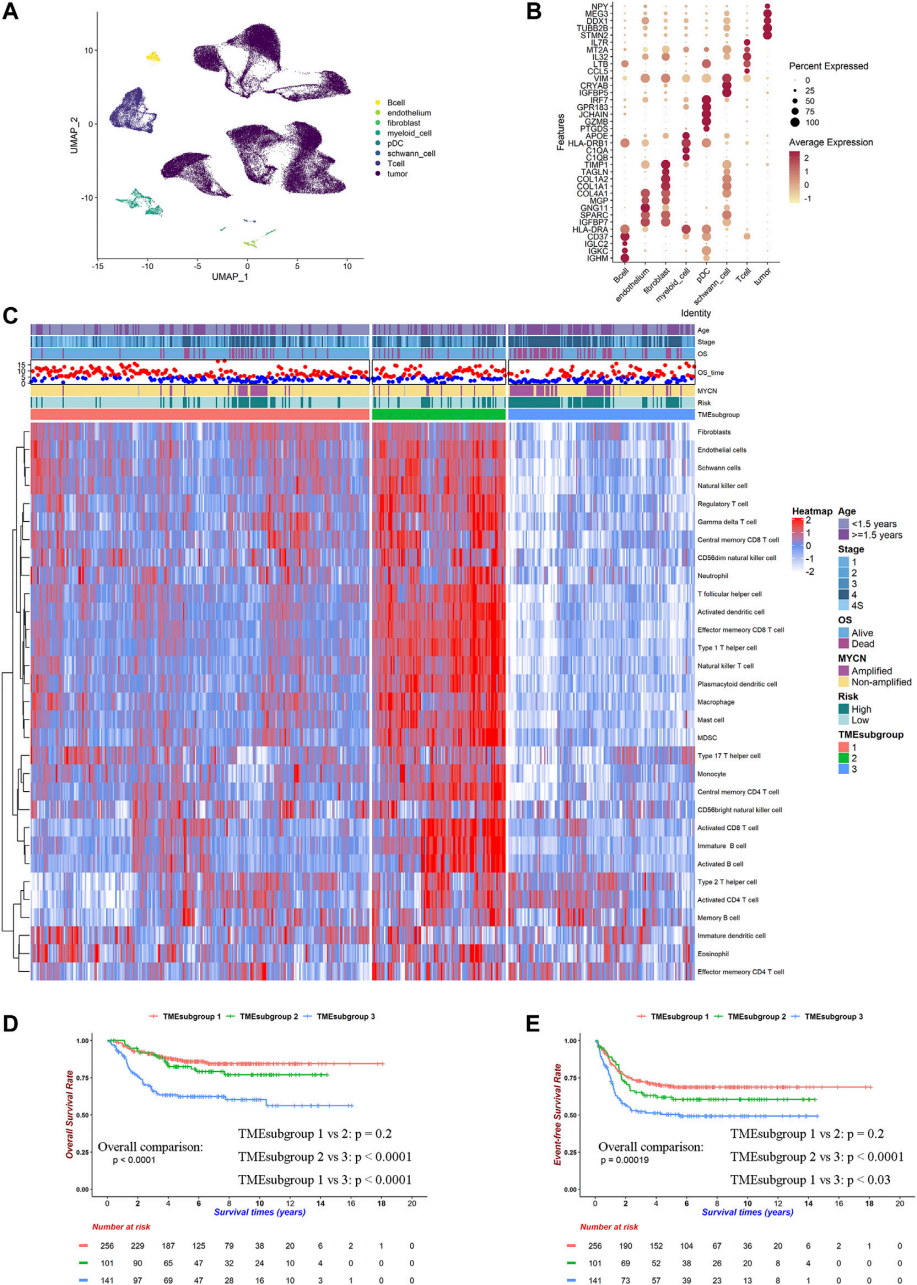
TME landscape of NB. Cell type markers were identified by single-cell RNA-seq and works of literature. Relative proportion of cells in 498 patients was calculated by ssGSEA method in SEQC cohort. **(A)** UMAP plot of filtered cells in six patients' single-cell RNA-seq data. **(B)** Dot plot of top 5 DEGs for selected cell types. **(C)** Heatmap for inferred relative cell proportions in SEQC cohort. Red indicates high, and blue indicates low proportion. **(D)** Kaplan–Meier curves of overall survivals for TMEsubgroups. Differences in survival rates were examined by log-rank test with a *p* < 0.0001 among these three subgroups. **(E)** Kaplan–Meier curves of event-free survivals for TMEsubgroups. Differences in survival rates were examined by log-rank test with a *p* = 0.00019 among these three subgroups.

We first evaluated a TME reference gene set from the single-cell RNA-seq data. A total of 83,867 cells from six patients, composed of B cells, T cells, pDC cells, myeloid cells, endothelial cells, fibroblasts, Schwann cells, and neuroendocrine tumor cells, were included in the analysis (Supplementary Figure S1A). We noticed a broad overlap between myeloid cells and tumor cells in clusters 1, 4, 9, 10, and 12 (Supplementary Figure S1B). We only retained myeloid cells in clusters 12, 14, and 20 and removed tumor cells in cluster 12 according to examine the expression of CD14 and FCER1G genes (Supplementary Figure S1C). Tumor-infiltrating immune cell estimation methods are highly relied on the actual presence of those cells in samples (Sturm et al., 2019). We detected CD3+FOXP3+ cells in one of the subsets of T cells, which laid the foundation for counting Tregs in bulk samples (Supplementary Figure S1D). Now, a re-annotated TME landscape of NB was delineated (Figure 1A). To construct an NB reference cell marker list, some genes must be added or discarded on the gene list from Charoentong et al. (2017). To this end, we performed the DEG analysis on each cell type and identified markers for them as the DEGs with the top log (fold-change) between the specific cell type and others (Figure 1B; Supplementary Table S2). Because the original gene list did not contain endothelial cells, fibroblasts, and Schwann cells, the top 20 markers of those cell types were appended to the list. Some tumor markers were also shown up in the list, which would make noises for inferring cell abundances. We removed all of them to precisely manifest the TME in bulk samples. Finally, a gene set made for NB was conducted (Supplementary Table S3).

### Tumor Microenvironment Landscape Characterized Three Prognostic Subgroups

We calculated the relative cell proportions on 498 NB samples from the SEQC cohort based on the altered gene list obtained by the single-cell analysis discussed earlier (Figure 1C). Patients were unsupervised clustered into three subgroups, which was determined by the consensus clustering method and was named “TMEsubgroup” (Supplementary Figure S2). TMEsubgroup3 lacked immune cell infiltrations except for the effector memory CD4^+^ T cells, activated CD4^+^ T cells, Th2 cells, immature DCs, eosinophils, and memory B cells. On the other hand, TMEsubgroup1 and 2 exhibited extensive penetrations of diverse cells compared with TMEsubgroup3 (all cell types except for the cell types mentioned earlier, Kruskal–Wallis rank-sum test, *p* < 0.0001). TMEsubgroup2 evinced high levels of the antitumor immunity (activated CD8^+^ T cells, NK cells, and NKT cells), pro-tumor immunity (Treg, MDSC, and Th2 cells), and stromal cells (endothelial cells, fibroblasts, and Schwann cells), which indicated that both cytolytic and inhibitory immune activities were activated in these patients. Similar results were obtained by five different algorithms (Supplementary Figures S3A–E). These TMEsubgroups discriminated by disparate cell infiltrations were prognostic in overall survivals and event-free survivals (log-rank test, *p* < 0.0001 and *p* = 0.00019, respectively, Figures 1D,E). However, TMEsubgroup1 and 2 did not significantly diverge in overall survivals and event-free survivals (log-rank test, *p* = 0.2 and *p* = 0.2, respectively, Figures 1D,E).

### Distinct Immune Cells Contributed Unequally to These Three TMEsubgroups

Next, we aimed to unravel the underlying immune mechanisms of these genes contributing to patient outcomes. We noticed that *MYCN* were amplified more in TMEsubgroup3 (11.46 *vs*. 9.00 *vs*. 38.57%, Chi-square test: *p* < 0.0001, Figure 1C). *MYCN*-amplified patients had high levels of activated CD4^+^ T cells, effector memory CD4^+^ T cells, and Th2 cells (Kruskal–Wallis rank-sum test, *p* < 0.0001, Supplementary Figure S3F). Instead, various types of CD8^+^ T cells, which were vital for antitumor activities, were enriched in *MYCN*-nonamplified samples. These results suggested that immune cells were relatively inadequate in TMEsubgroup3.

To validate that the immune cells were absent in the high-risk group, we conducted IHC profiling on four stage 1 and four stage 4 samples. CD3^+^ T cells were enriched in the tumors of stage 1 samples (Supplementary Figure S4A) compared with those of stage 4 (Supplementary Figure S4B). Similar results were found that CD8^+^ T cells infiltrated into tumors of stage 1 (Supplementary Figure S4C), whereas seldom did they exist in stage 4 patients (Supplementary Figure S4D). Quantitative analysis also revealed that CD3^+^ T and CD8^+^ T cell proportions were relatively higher in 12 regions from stage 1 patients (Supplementary Figures S4E, F, Student *t*-test, *p* < 0.0001 and *p* = 0.0012, respectively). These results demonstrated that the high-risk patients, corresponding to TMEsubgroup3, owned rare immune cells to activate immune responses.

We noticed that the number of immature DCs was negatively correlated with multiple cell types, including activated CD8^+^ T cells (Pearson's correlation efficiency: −0.265, *p* < 0.0001, Supplementary Figure S4G). DCs could present tumor-associated-antigens on MHC 1 molecules to activate CD8^+^ T cells and promote immunity Wculek et al., 2020. Previous reports indicated that an increase of immature DCs could lead to anergy or deletion of activated T cells (Melief, 2008).

We also examined the contributions of each type of cell to clinical outcomes. Activated CD4^+^ T cells, monocytes, Th2 cells, and Th17 cells were prognostic in all TMEsubgroups (*p* < 0.05, Supplementary Figure S4H; Supplementary Table S4), which were in concordance with previous reports (Cheung and Dyer, 2013). Higher MDSC levels heralded poor survivals in TMEsubgroup1 and 2, which might be due to their negative regulation of immune responses (Heczey et al., 2020; Ladenstein et al., 2018). Besides, we observed that stromal cells, such as endothelial cells, fibroblasts, and Schwann cells, were beneficial for patients in the whole SEQC cohort, where HR ranged from 0.602 to 0.804 (Supplementary Table S4). It was illustrated that only neuroendocrine tumor cells gained *MYCN* copies instead of all cells in the NB microenvironment in previous single-cell analyses (Dong et al., 2020). Our results further clarified that stromal cells might not be the crucial factor for NB progression. Overall, we demonstrated that the aggressive tumor subtype was accompanied by immune suppression, which leads to tumor progression.

### Gene Profiling Represented the Tumor Microenvironment Landscape

**FIGURE 2:**
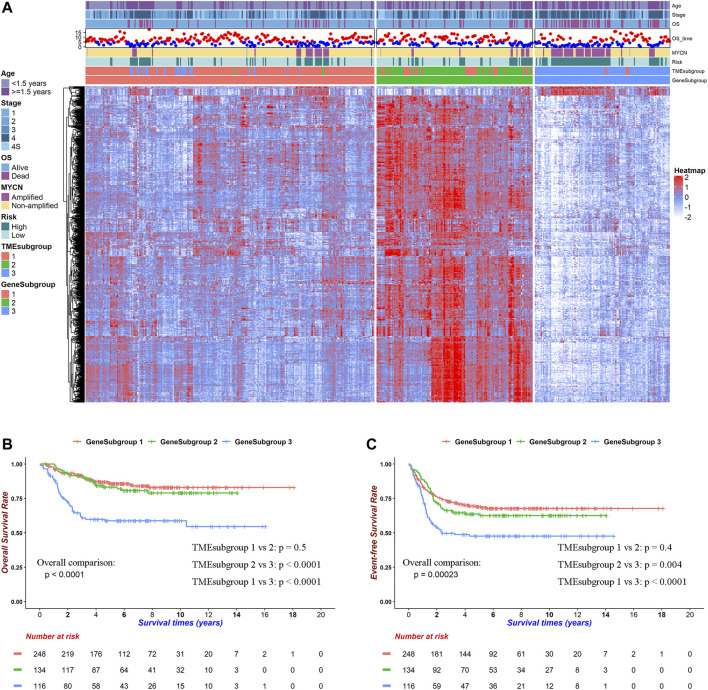
Transcriptome background within TME landscape. DEGs among TMEsubgroups clustered patients into three prognostic GeneSubgroups. **(A)** Heatmap for 786 DEGs among TMEsubgroups. Red indicates high, and blue indicates low expression. **(B)** Kaplan–Meier curves of overall survivals for GeneSubgroups. Differences in survival rates were examined by log-rank test with a *p* < 0.0001 among these three subgroups. **(C)** Kaplan–Meier curves of overall survivals for GeneSubgroups. Differences in survival rates were examined by log-rank test with a *p* = 0.00023 among these three subgroups.

We first performed pairwise DEG analysis on these three TMEsubgroups (Supplementary Table S5). A total of 786 genes were selected after strict cutoffs with the absolute of log2(fold-change) that were greater than 1.4 and an adjusted *p*-value below 0.05. These DEGs divided patients into three clusters, which were determined by the consensus clustering method and were named “GeneSubgroup” (Figure 2A; Supplementary Table S5). The matching rates of TMEsubgroup to GeneSubgroup were 86.69, 73.13, and 95.69% for subgroups 1, 2, and 3, respectively (i.e., 86.69, 73.13, and 95.69% samples of GeneSubgroup1, two and three were from TMEsubgroup1, 2, and 3, respectively), showing the association between the TME landscape and the gene expressions atlas (Chi-square test: *p* < 0.0001). Therefore, we could depict the TME landscape at the transcriptome level.

We noticed that GeneSubgroup3 owned more aged (30.24, 39.55, and 56.03% for ages greater than 1.5 years in GeneSubgroup1, 2, and 3, Chi-square test: *p* < 0.0001) and *MYCN*-amplified (11.43, 9.02, and 45.22% in GeneSubgroup1, 2, and 3, Chi-square test: *p* < 0.0001) patients, and these were known risk factors for NB outcomes (Figure 2A). Undeniably, three GeneSubgroups were prognostic in overall survivals and event-free survivals (Figures 2B,C, both log-rank test: *p* < 0.0001). However, GeneSubgroup1 and 2 did not differ in survivals significantly (Figures 2B,C, log-rank test: *p* = 0.5 and 0.4, respectively). Thus, the GeneSubgroups showed analogous clinical characteristics and outcomes with the TMEsubgroups.

### GeneSubgroup3 Was Prone to Carcinogenesis

**FIGURE 3:**
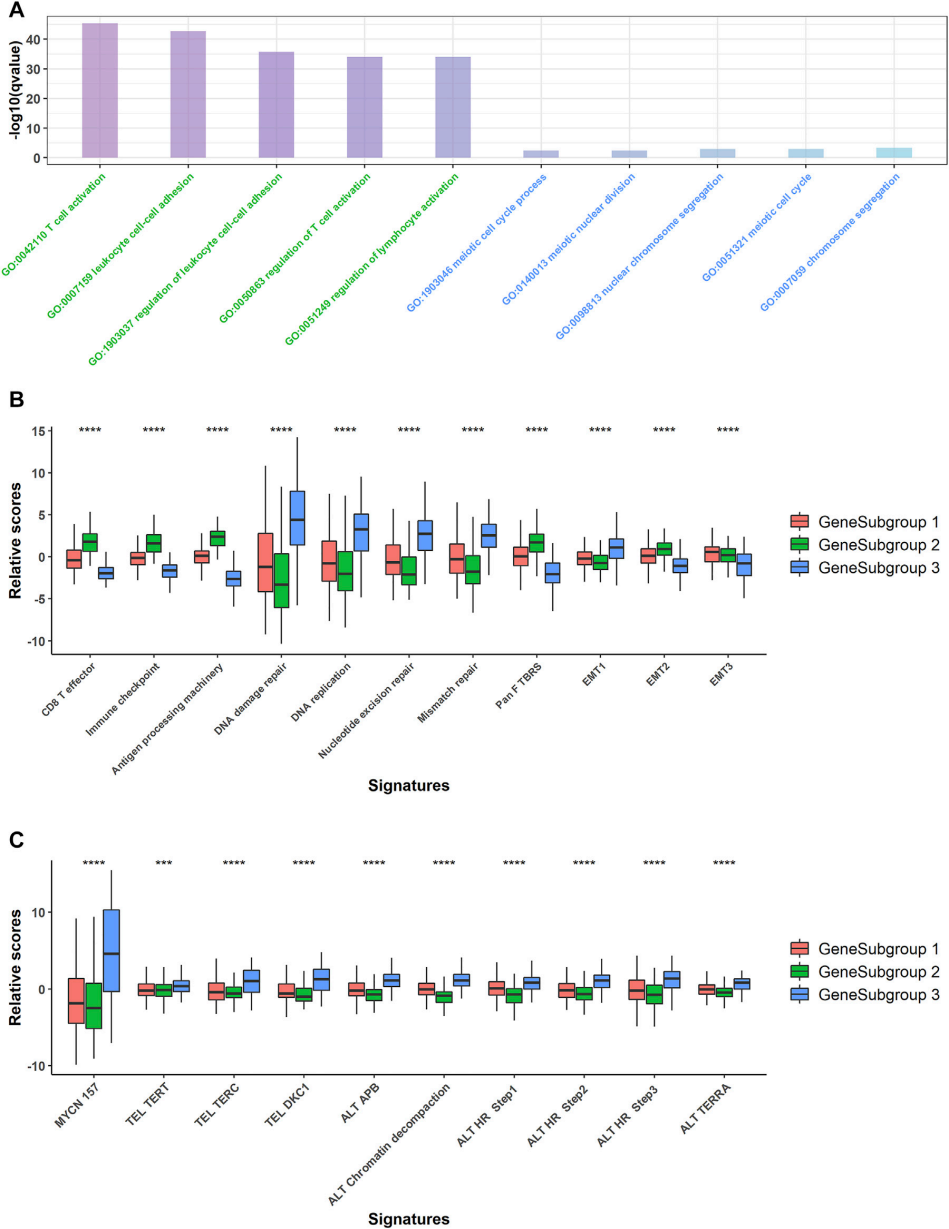
Pathway analysis for GeneSubgroups. **(A)** Downregulated genes in GeneSubgroup3 enriched in immune-related pathways (left five terms, green) and upregulated genes enriched in cell cycle related pathways (right five terms, blue). **(B)** Boxplots of relative scores of *MYCN*-related pathway signatures. *p*-values were generated through Kruskal–Wallis rank-sum tests and labeled above each boxplot with asterisks (ns: *p* > 0.05, *p < 0.05, **p < 0.01, ***p < 0.001, ****p < 0.0001). **(C)** Boxplots of relative scores of carcinogenesis pathway signatures. *p*-values were generated through Kruskal–Wallis rank-sum tests and labeled above each boxplot with asterisks (ns: *p* > 0.05, *p < 0.05, **p < 0.01, ***p < 0.001, ****p < 0.0001).

We conducted GO-enrichment analysis for up- and downregulated genes in GeneSubgroup3 compared with GeneSubgroup 2 and found that immune responses decreased in GeneSubgroup3 (Figure 3A, left five GO terms). On the other hand, cell-cycle-related pathways took vital roles in GeneSubgroup3 (Figure 3A, right five GO terms). This was in line with the patterns in the TMEsubgroup, where the poorest survived subcluster displayed a dearth of immune cells and rapid cell division.

Thus, we compared the proven oncogenesis signals among the GeneSubgroups. The GeneSubgroup3 showed high scores in an *MYCN*-downstream signature and nine telomere maintenance mechanism-related pathways; all of these pathways were the causes of NB tumorigenesis and progression (19, 20) (Kruskal–Wallis rank-sum test: *p* < 0.0001, Figure 3B). Generally, tumor oncogenesis is associated with mismatch repair, in which GeneSubgroup3 outstripped the other two groups (Kruskal–Wallis rank-sum test: *p* < 0.0001, Figure 3C). In addition, CD8^+^ effector and antigen processing signals decreased in GeneSubgroup3, making them hard to trigger an immune response. In two of three EMT-associated signatures, GeneSubgroup3 scored less, indicating that the epithelial–mesenchymal transition might not be the main source of aggressive phenotypes (Kruskal–Wallis rank-sum test: *p* < 0.0001, Figure 3C). These data suggested that patients in GeneSubgroup3 were susceptible to being affected by classical tumor growth pathways.

### Lack of Immune Cells Contributed to Extrinsic Immune Escape in GeneSubgroup3

**FIGURE 4:**
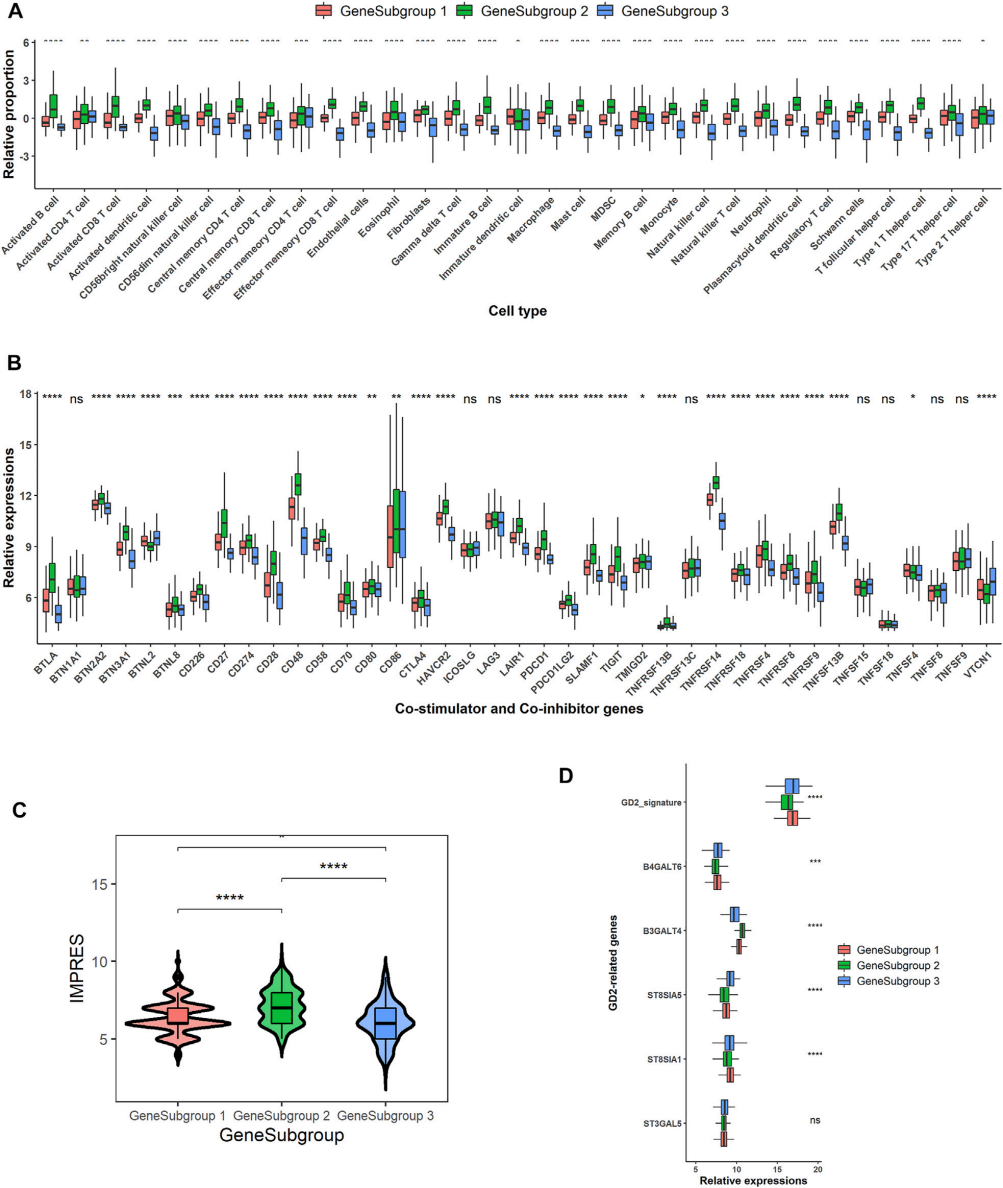
Potential extrinsic and intrinsic immune escape mechanisms for GeneSubgroup3. **(A)** Boxplots of relative cell proportions among GeneSubgroups. Thick line represents median value. Bottom and top of boxes are 25th and 75th percentiles, respectively. Whiskers encompass 1.5 times interquartile range. *p*-values were generated through Kruskal–Wallis rank-sum tests and labeled above each boxplot with asterisks (ns: *p* > 0.05, *p < 0.05, **p < 0.01, ***p < 0.001, ****p < 0.0001). **(B)** Boxplots of co-stimulator and co-inhibitor gene expressions among GeneSubgroups. *p*-values were generated through Kruskal–Wallis rank-sum tests and labeled above each boxplot with asterisks (ns: *p* > 0.05, *p < 0.05, **p < 0.01, ***p < 0.001, ****p < 0.0001). **(C)** Violin plots of IMPRES among GeneSubgroups. *p*-values were generated through pairwise Wilcoxon rank-sum tests and labeled above each boxplot with asterisks (ns: *p* > 0.05, *p < 0.05, **p < 0.01, ***p < 0.001, ****p < 0.0001). **(D)** Boxplots of GD2-related gene expressions and GD2 signature scores. *p*-values were generated through Kruskal–Wallis rank-sum tests and labeled above each boxplot with asterisks (ns *p* > 0.05, *p < 0.05, **p < 0.01, ***p < 0.001, ****p < 0.0001).

The extrinsic immune escape theory suggested that some of the TME components assisted and decided the immune escape of tumor cells (Mohme et al., 2017; Schreiber et al., 2011). First, the lack of immune cells, especially the cytolytic cells, could be the most devastating event for the immune system. All the TME ingredients except for activated CD4^+^ T cells, effector memory CD4^+^ T cells, immature DCs, and Th2 cells were significantly lower in GeneSubgroup3 (Kruskal–Wallis rank-sum test except for cells discussed earlier: *p* < 0.0001, Figure 4A). Secondly, Tregs and MDSCs could suppress innate immune functions (Veglia et al., 2018; Sharabi et al., 2018). We found that Tregs and MDSCs slumped in GeneSubgroup3 (Kruskal–Wallis rank-sum test: both *p* < 0.0001, Figure 4A). This indicated that such cells might not be the source of immune-silent states in GeneSubgroup3. Thirdly, high concentrations of immunoinhibitory cytokines, such as IL-10, would inhibit the proliferation and differentiation of T cells in GeneSubgroup2 instead of GeneSubgroup3 (Kruskal–Wallis rank-sum test: *p* < 0.0001 Supplementary Figure S6A). In GeneSubgroup3, patients were short of various types of chemokines (such as CCR1) and tumor necrosis factors as well as their superfamily members, which were crucial for attracting immune cells and inducing tumor apoptosis (Supplementary Figures S6B, C) (Cheng and Jack, 2008; Nagarsheth et al., 2017; Dostert et al., 2019).

### GeneSubgroup3 Would Benefit From Anti-GD2 Therapies Instead of Anti-PD1 or Anti-CTLA4

The immune intrinsic escape mechanism emphasized the tumor immunogenicity and immune checkpoint molecule expression in antitumor immunity (Schreiber et al., 2011; Mohme et al., 2017). The HLA molecules were downregulated in GeneSubgroup3, and this could result in the loss of antigen presentation on the tumor cell surface (Prigione et al., 2004) (Supplementary Figure S6D). High expressions of immune checkpoint genes, e.g., CTLA-4 and PD-1, would aid tumors to escape from immune surveillance (Ribas and Wolchok, 2018), and the immune checkpoint therapies prolonged overall survival in many cancer types, including advanced melanoma and urothelial cancers (Robert et al., 2015; Horn et al., 2018; André et al., 2020). GeneSubgroup3 exhibited the lowest expressions of PDCD1 (PD-1) and CD274 (PD-L1), suggesting that these patients might not be profitable for anti-PD-1 or anti-PD-L1 therapies (Figure 4B). A higher value of IMPRES, a well-constructed immune-therapy predictor, could forecast response status before the administration of drugs. GeneSubgroup3 scored low, implying the minimal effect of using those remedies (median: 6, 8, and 6; Wilcoxon rank-sum test: *p* < 0.0001, *p* < 0.0001, and *p* = 0.01 for GeneSubgroup1 to GeneSubgroup2, GeneSubgroup2 to GeneSubgroup3, and GeneSubgroup1 to GeneSubgroup3, respectively, Figure 4C).

GD2 antibodies have been in clinical trials and displayed outstanding effects (Robert et al., 2015; Horn et al., 2018; André et al., 2020). We scrutinized GD2 synthases expressions and calculated GD2+ predictor scores, the sum of *ST8SIA1* and *B4GALNT1*, as the previous report (21). We found that GeneSubgroup3 got higher scores than GeneSubgroup2 (median: 16.97 and 16.35; Wilcoxon rank-sum test: *p* < 0.0001) but not differed from GeneSubgroup1 (median: 16.97 and 16.89; Wilcoxon rank-sum test: *p* > 0.05), suggesting that GeneSubgroup3, rather than GeneSubgroup2, might be favorable for using anti-GD2 regimes (Figure 4D). However, as the GD2 protein is mainly expressed on tumor cells, the higher GD2 levels in GeneSubgroup3 might be attributed to higher tumor purities, and more validation is needed to examine the average GD2 levels between groups. These data demonstrated that the immune-cold patients were not able to trigger antigen presentation processes *via* HLA molecules. They also could not benefit from anti-PD-1 and anti-PD-L1 therapies.

### TMEscore was Prognostic for Neuroblastoma and Other Cancers

**FIGURE 5:**
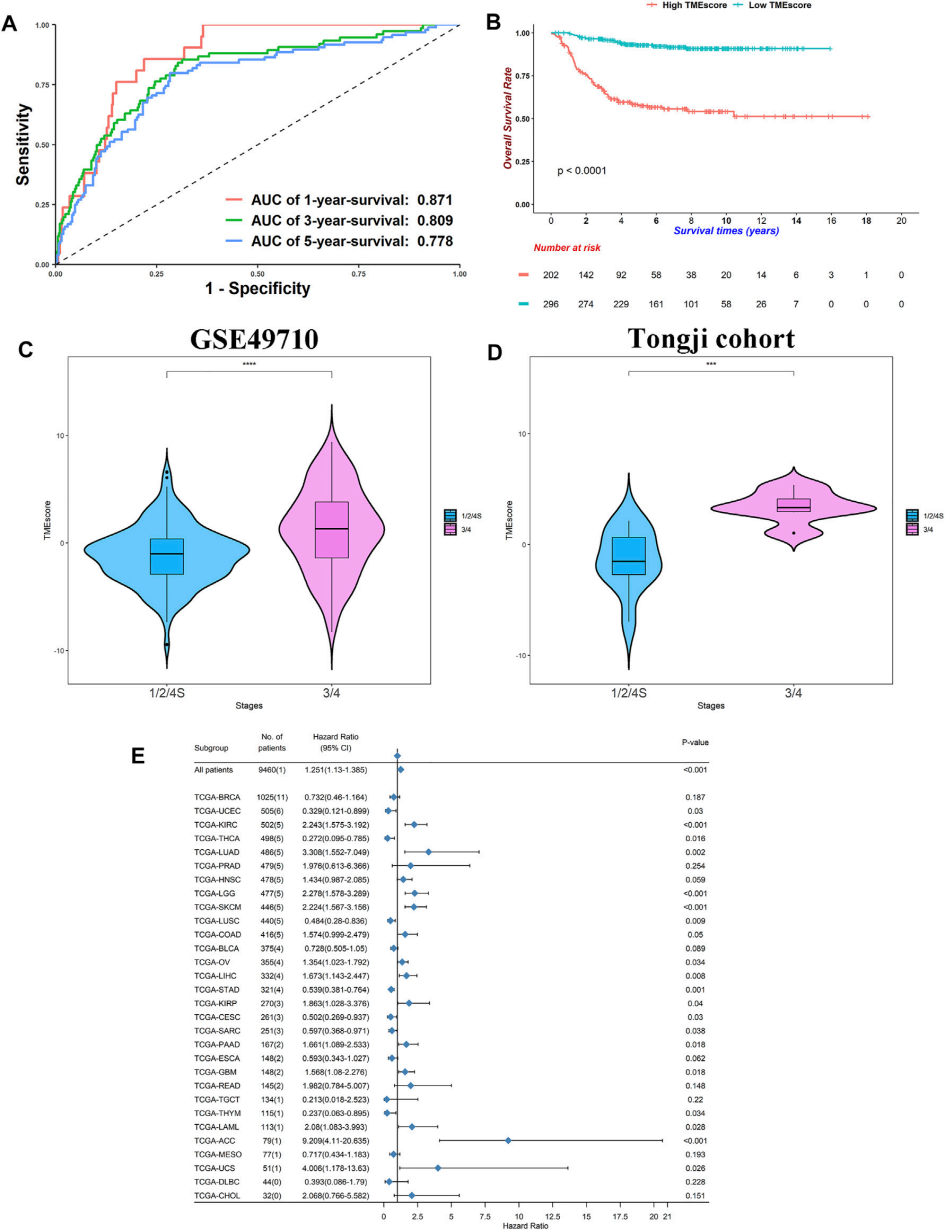
TMEscore was prognostic in NB and other cancers. **(A)** Receiver operating characteristic curves for 1-, 3-, and 5-year survival in SEQC cohort. **(B)** Kaplan–Meier curves of overall survivals for high- and low-TMEscore subgroups. Differences in survival rates were examined by log-rank test with a *p* < 0.0001. **(C)** Violin plot of TMEscores between stage 1/2/4S and stage 3/4 in SEQC cohort. *p*-values were generated through Kruskal–Wallis rank-sum tests and labeled above each boxplot with asterisks (ns: *p* > 0.05, *p < 0.05, **p < 0.01, ***p < 0.001, ****p < 0.0001). **(D)** Violin plot of TMEscores between stage 1/2/4S and stage 3/4 in Tongji cohort. *p*-values were generated through Kruskal–Wallis rank-sum tests and labeled above each boxplot with asterisks (ns: *p* > 0.05, *p < 0.05, **p < 0.01, ***p < 0.001, ****p < 0.0001). **(E)** Forest plot of hazard ratios for 30 TCGA projects. *p*-values were calculated by Cox regressions.

To construct a prognostic signature, we conducted univariable Cox regressions and selected 48 genes whose adjusted *p*-values were <0.05. According to their HR, we divided them into groups A and B (Supplementary Table.S6). We calculated ScoreA and ScoreB described in the Method Section and defined their subtraction as the “TMEscore” (Supplementary Table.S7). The TMEscore showed prognostic values, where area under the ROC curve (AUC) values at 5-year survival reached 0.778, 0.750, 0.720, and 0.705 in the SEQC, TARGET, E-MTAB-179, and E-MTAB-8248 cohorts, respectively (Figure 5A; Supplementary Figures S7A–C). Except for the E-MTAB-8248 cohort, all datasets owned AUCs of ROCs greater than 0.7 at 1-, 3-, and 5-year survival (Figure 5A; Supplementary Figures S7A–C). This indicated that our signature is predictive and robust. We then divided patients into high- and low- TMEscore groups. The high-TMEscore group survived significantly shorter than the low-TMEscore group in four datasets (log-rank test: all *p* < 0.0001 for the SEQC, TARGET, E-MTAB-179, and E-MTAB-8248 cohorts, respectively, Figure 5B; Supplementary Figures S7D–F).

Of note, the TMEscore increased in stage 3/4 patients relative to stage 1/2/4S (median: 1.011 *vs*. −1.3249, Wilcoxon rank-sum test: *p* < 0.0001, Figure 5C). We did high-throughput RNA-sequencing on six stage 3/4 samples and 10 stage 1/2 samples. Using the same groups of genes and algorithms, we found a similar result in our Tongji cohort (median: 3.329 *vs*. −1.5182, Wilcoxon rank-sum test: *p* < 0.001, Figure 5D; Supplementary Table S8). These results demonstrated that the elevated TMEscore was associated with aggressive phenotype and decreased survival rates.

We calculated the TMEscore of all patients in the TCGA pan-cancer project (Supplementary Table S9; Figure 5E). The TMEscore was prognostic in 20/30 cancer types at *p* < 0.05, indicating that the TMEscore could be utilized to predict survival in these cancers. Conspicuously, high TMEscore heralded poor outcomes in the kidney renal clear cell carcinoma, brain lower-grade glioma, SKCM, and adrenocortical carcinoma at *p* < 0.001 level (HR: 2.243, 2.278, 2.224, and 9.209, respectively). These data indicated that NB might share similar carcinogenesis, progression mechanisms, and TME cross-talk with these cancers because NB was derived from the sympathetic nervous systems and plentiful cases were found at the adrenal glands (Maris et al., 2007). However, no reports or animal experiments have linked NB with other cancers at the TME level, and further studies are needed. Overall, we successfully generated a signature that could predict survivals for NB and other cancers.

### TMEscore Could be a Predictor of Immunotherapy Response

**FIGURE 6:**
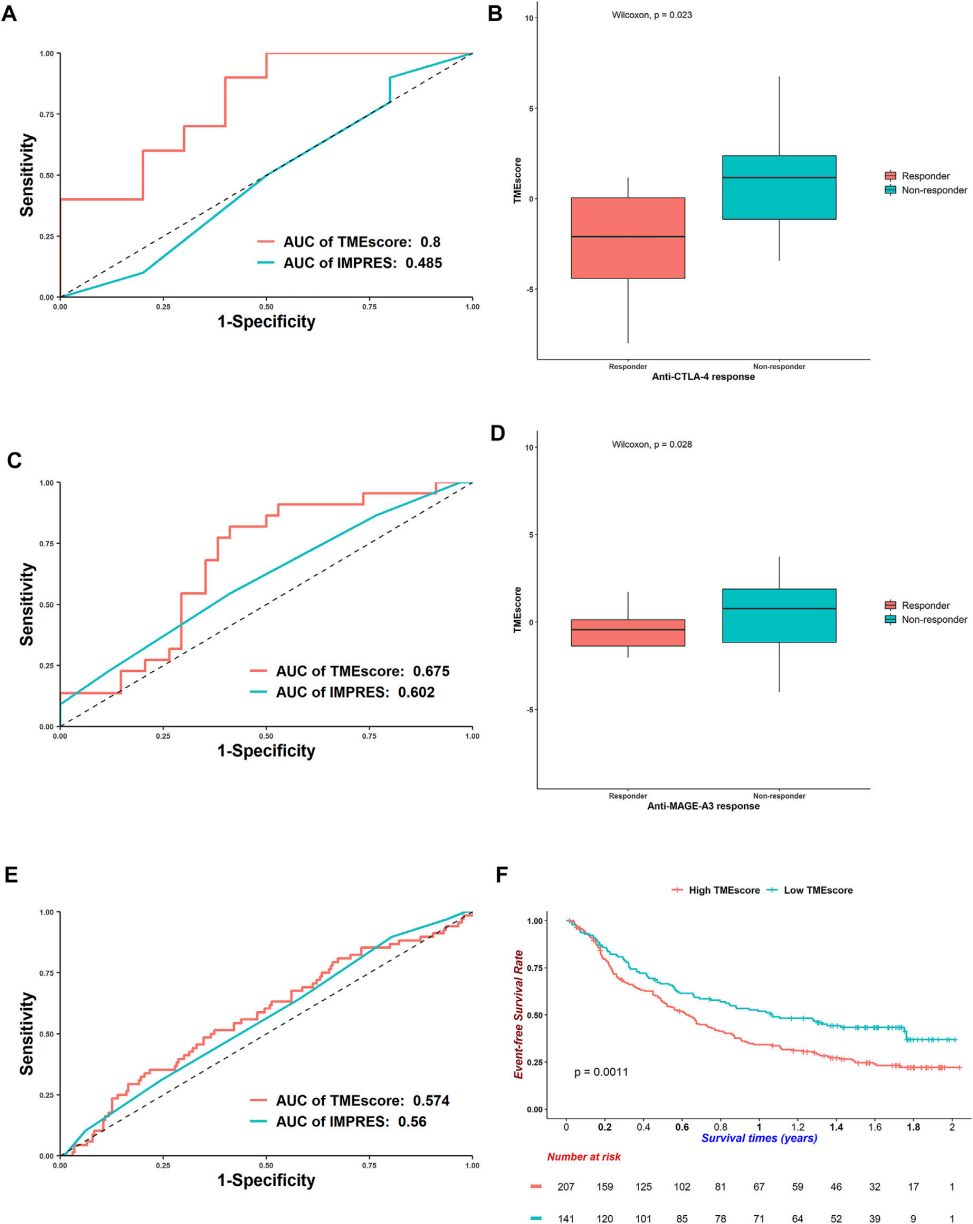
Efficacy of TMEscore to predict responses for immunotherapies. **(A)** Receiver operating characteristic curves of TMEscore and IMPRES to predict responses for anti-CTLA-4 mouse model. **(B)** Boxplots of TMEscore between responders and nonresponders for anti-CTLA-4 mouse model (Wilcoxon rank-sum test: *p* = 0.023). **(C)** ROC curves of TMEscore and IMPRES to predict responses for anti-MAGE-3 melanoma patients. **(D)** Boxplots of TMEscore between responders and nonresponders for anti-MAGE-3 melanoma patients (Wilcoxon rank-sum test: *p* = 0.028). **(E)** ROC curves of TMEscore and IMPRES to predict responses for anti-PD-L1 urothelial cancer patients. **(F)** Kaplan–Meier curves of overall survivals for urothelial cancer patients treated with anti-PD-L1. Differences in survival rates were examined by log-rank test with a *p* = 0.0011.

The TMEscore was derived from the microenvironment niche inside tumors where immune checkpoint drugs took effect (Quail and Joyce, 2013). We proposed that the TMEscore could represent the immune states of patients. A high TMEscore meant the low expression of immune-related genes and thus poor responses for immunotherapies. Indeed, for the anti-CTLA-4-treated AB1-HA mouse model, the TMEscore reached an AUC of 0.8, whereas a well-established immunotherapy response predictor, the IMPRES, only gained 0.485 on this dataset (Figure 6A). Responders received a lower TMEscore compared with nonresponders, which was in concordance with our hypothesis (Wilcoxon rank-sum test: *p* = 0.023, Figure 6B). Considering that these results were attained in a mouse model, we further analyzed other datasets. For the anti-MAGE-3 melanoma patients, the TMEscore got an AUC of 0.675 (Figure 6C), and points for responders were also lower than those for nonresponders (Wilcoxon rank-sum test: *p* = 0.028, Figure 6D).

For the urothelial cancer patients treated with anti-PD-L1, the AUC of TMEscore decreased, and the TMEscore performed better than IMPRES (Figure 6E). Besides, the TMEscore could divide patients into prognostic groups (log-rank test: *p* = 0.0011, Figure 6F). The TMEscore was also prognostic for the anti-PD-1 melanoma patients (log-rank test: *p* = 0.0029, Supplementary Figure S8A); however, it could barely predict responses (AUC = 0.503, Supplementary Figure S8B). For the TCGA-SKCM patients who received various immunotherapies, a higher TMEscore was correlated with worse survivals (log-rank test: *p* = 0.005, Supplementary Figure S8C). Although the AUC of TMEscore was 0.711 (Supplementary Figure S8D), responders got higher scores than nonresponders, which was opposite with the trends discussed earlier (Wilcoxon rank-sum test, *p* = 0.021, Supplementary Figure S8E). This is partly because patients were administered diverse remedies. Overall, the TMEscore could predict responses for multiple immunotherapies.

### TMEIndex Rather Than Children’s Oncology Group Risk Classification Predicated Neuroblastoma Outcomes

We conducted a meta-analysis to assess correlations between the TMEscore and clinical covariates. Higher TMEscores were hazardous in both <1.5 years and older subgroups (HR = 8.241 and 2.346, respectively, both *p* < 0.001, Supplementary Figure S9A). We saw similar results in INSS stage stratifications (HR = 7.003 for stage 1/2/3/4S and HR = 2.379 for stage 4, both *p* < 0.001, Supplementary Figure S9A). However, we did not see this trend in the *MYCN* subgroup analysis (HR = 1.125, *p* = 0.583 in *MYCN*-amplified patients, Supplementary Figure S9A). The TMEscore, along with age, *MYCN* status, sex, INSS stage, and COG risk, was a risk factor of NB patients (Supplementary Figure S9B). The TMEscore became significant in multivariable Cox regression analysis regardless of other covariates, indicating that it could be an independent survival predictor (HR = 2.578, *p* < 0.001, Supplementary Figure S9C).

We further constructed a nomogram, which was convenient and effective in practice, based on the TMEscore, age, *MYCN* status, sex, and INSS stage (Supplementary Figure S10A). The TMEIndex, which was counted as the total point a patient received in the nomogram, agreed with 5-year-survival outcomes better than the TMEscore (C-index: 0.843 and 0.730 for TMEIndex and TMEscore, respectively, Supplementary Table S7; Supplementary Figure S10B). The TMEIndex gained the highest AUC in five predictors, including the COG risk system, suggesting that our TMEIndex was helpful for predicting outcomes (Supplementary Figure S10C). We compared the AUC between the TMEscore and COG risk and TMEIndex and COG risk using nonparametric estimation in the timeROC package. The TMEscore did not differ from COG risk; however, the TMEIndex got higher AUC, suggesting that there was an additional benefit in TMEIndex beyond the clinical–histological COG-risk classification system (TMEscore *vs*. COG risk: *p* = 0.0294 and TMEIndex *vs*. COG risk: *p* < 0.0001, where adjusting *p* = 0.05/2 = 0.025 when conducting multiple comparisons). Finally, basic information about patients in our research was summarized in the alluvial diagram (Supplementary Figure S10D).

## Discussion

Armed with four large independent NB cohorts, we deciphered TME inside NB tumors and explored their interactions within TME as well as their correlations with clinical features and outcomes. We first curated a cell type marker list specific for NB TME by utilizing single-cell RNA-seq data. Based on the gene list, we inferred relative cell proportions in the largest NB cohort, the SEQC cohort, to cluster patients into three prognostic TMEsubgroups and used DEGs to cluster patients into GeneSubgroups. TMEsubgroup/GeneSubgroup1 and 2 were so-called immune-hot tumors, whereas subgroup 3 were immune-cold. We constructed TMEscore to predict outcomes in four NB cohorts and verified that higher INSS stages got higher scores in the Tongji cohort by 16 high-throughput RNA-seq samples. We further expanded TMEscore to 30 types of cancers in TCGA database, and 20 of which had *p*-values lower than 0.05 in Cox regressions. In addition, TMEscore was excellent at predicting immunotherapy responses. Finally, we created a nomogram-based TMEIndex to aid physicians in determining remedies.

Our TME inferring results were of high authenticity and reliability. Quantification of tumor-infiltrating cells from transcriptomics data could be distinguished in three methods: single-cell RNA-sequencing (scRNA-seq) approaches, marker-gene-based approaches, and deconvolution-based approaches (Robert et al., 2015; Horn et al., 2018; André et al., 2020). However, scRNA-seq remains expensive and time-consuming, which is not suitable for clinical use. The latter two ways rely on the selection of the reference profiles (Avila Cobos et al., 2018; Finotello and Trajanoski, 2018). For the marker-gene-based approaches, gene sets are usually derived from targeted transcriptomics studies characterizing each cell type and/or from comprehensive literature search and experimental validation (Avila Cobos et al., 2018; Finotello and Trajanoski, 2018). For the deconvolution-based approaches, cell types and cell type markers must be presented in the mixture, and true composition must be known (Avila Cobos et al., 2018; Finotello and Trajanoski, 2018). A well-known deconvolutional method, *cibersort*, estimates cell abundances based on the 22 peripheral blood mononuclear cell types microarray data (https://cibersort.stanford.edu/). However, immune cells in NB TME would be quite different from what existed in circulations. To address these issues, we adopted the scRNA-seq + ssGSEA pipeline as described in the Methods Section, which owned the following advantages. First, we only used small amounts of samples for scRNA-seq exploration and ciphered cell proportions in bulk RNA-seq samples, which is economical for clinical cases. Second, we confirmed the existence of rare cell types, e.g., Tregs, which laid the foundations for calculating cell proportions. Third, we removed tumor cell markers from the gene list. Solid tumor cells occupy a large proportion of the tumor tissue, and these genes would exaggerate inferred cell abundances because they are often expressed higher in the bulk sample. We believed that this pipeline would give a precise and concise TME compendium for NB.

In our analysis, three prognostic TMEsubgroups were identified. To investigate these TMEsubgroups at the transcriptome level, we mapped them into corresponding GeneSubgroups. GeneSubgroup1 and 2 had higher proportions of immune cells compared with GeneSubgroup3 and immune-related pathways. Moreover, GeneSubgroup2 retained high immune suppressive cell types such as MDSCs and Tregs. Also, immune checkpoint genes expressed more in GeneSubgroup1 and 2. This indicated that GeneSubgroup1 and 2 could be categorized as immune-hot or inflamed tumors due to their presence of infiltrating immune cells, high density of IFN-γ-producing CD8^+^ T cells, and expression of PD-L1 (Hegde et al., 2016). Immune-hot tumors responded favorably to immune checkpoint inhibition in many cancers, and thus, GeneSubgroup1 and 2 could benefit from these therapies (Hegde et al., 2016). Nonetheless, TMEsubgroup/GeneSubgroup2 owned a large proportion of fibroblasts, endothelial cells, and Schwann cells, which is hardly reported in previous TME studies of NB. In a pan-cancer study, the immune-enriched and fibrotic subgroup, which was similar to our TMEsubgroup/GeneSubgroup2, survived less than the immune-enriched but no fibrotic subgroup in melanoma patients and TCGA cohort with 8,024 patients (Bagaev et al., 2021). In this work, researchers found that the immune-enriched but no fibrotic subgroup had the highest mutation load. We hypothesized that TMEsubgroup/GeneSubgroup1 had more mutations to stimulate the immune system. In contrast, we found that GeneSubgroup1 had low expression of HLA genes. Thus, investigations about mutation load, neoantigen load, and antigen-presenting mechanisms in different clusters of NB patients are needed. On the other hand, GeneSubgroup3 was characterized by highly proliferating tumors and low expressions of MHC class I, just the same as the so-called immune-cold or non-inflamed tumors (Hegde et al., 2016). Pediatric malignancies are representative examples of non-inflamed tumors (Alexandrov et al., 2013). The 2-year risks of relapse for immune-hot and -cold patients were 10 and 80% in colorectal cancer, respectively (Camus et al., 2009). Indeed, TMEsubgroup/GeneSubgroup3 had significantly lower overall survival and event-free survival. Thus, we successfully defined immune subgroups analogy with other cancers. It is suggested that immune checkpoint inhibition could be administered to immune-hot tumors (Galon and Bruni, 2019). For the immune-cold samples, a combined therapy that enhances T cell responses and inhibits immune checkpoint signals is still controversial (Galon and Bruni, 2019). In our analysis, we demonstrated that GD2 signals were higher in immune-cold tumors. However, this phenomenon might be exaggerated by the higher tumor purities in these patients. We could not precisely calculate or estimate tumor purities in pediatric cancers because such investigations were rare. Thus, quantifications and comparisons of GD2 levels between high- and low-risk groups after sorting *EPCAM* + tumor cells are expected.

Researchers are keen on searching signatures that could predict survival and immunotherapy responses (Galon et al., 2013; Auslander et al., 2018). However, these studies often only considered immune-related genes. We first created two signatures, ScoreA and ScoreB, and combined them as TMEscore. ScoreA is a signature consisting of upregulated genes in TMEsubgroup2, which are mainly immune-related genes. In contrast, the ScoreB signature is composed of five hazardous genes (*MX2*, *FPR1*, *CEBPB*, *CLEC4G*, and *CPLX3*). One of the variants in *MX2* was associated with melanoma (Barrett et al., 2011). *FPR1* has been found to be overexpressed on some tumor cells and mediating antitumor immunity and metastasis (Wong et al., 2014). *CEBPB* pathways affected MDSCs and maintained tumor immunosuppression in triple-negative breast cancer (Li et al., 2018). These studies highlighted the importance of such genes in the cross-talks of TME and emphasized the need to include these genes into prognostic signatures. Our TMEscore gained high AUCs at 1-, 3-, and 5-year-survival in four independent cohorts. Also, patients with high and low TMEscores diverged in overall survival significantly. We sequenced six stage 3/4 and 10 stage 1/2 samples and validated that aggressive samples got higher scores. In 20/30 tumor types, TMEscore could predict survival, indicating that this model could be taken into account in clinical applications. This result also indicated that our signature reflects common pathways in the pan-cancer-wide field. Besides, TMEscore could foretell immunotherapy responses. We compared TMEscore with IMPRES, an immune checkpoint score system derived from NB and validated on over 10 anti-PD-1, anti-PD-L1, and anti-CTLA-4 datasets (Galon et al., 2013; Auslander et al., 2018). TMEscore outperformed IMPRES in three datasets, which shed light on predicting anti-CTLA-4 and anti-MAGE-3 responses in NB.

There are some limitations to this study. First, we only did IHC staining of CD3 and CD8 but did not conduct fluorescence-activated cell sorting, which is one of the gold standards for estimating cell content, to validate our self-designed pipeline (Petitprez et al., 2018). Second, we only follow-up 1-year survival in the Tongji cohort. Thus, we could not validate TMEscore for survival analysis. Third, we did not obtain sequencing data of immunotherapy trials for NB, and this resulted in the inability to test TMEscore in the prediction of responses in NB. Fourth, we inferred that high-risk NB patients might benefit from anti-GD2 therapies without clear evidence, and further investigations should be put forward. We hope these limitations can be resolved in the future.

In summary, we computed cell proportions in bulk samples using a self-curated gene list and identified three subgroups of NB patients. The patients with the poorest survival could benefit from anti-GD2 instead of anti-PD-1 drugs. We created TMEscore to predict survivals and constructed a nomogram-based TMEIndex, which could substitute the current COG risk system in clinical cases. We hope our analysis could pave the way for NB TME investigation and immunotherapy drug use in NB.

## Data Availability

The datasets presented in this study can be found in online repositories. The names of the repository/repositories and accession number(s) can be found in the article/Supplementary Material.
