# Supplementary material for: Tumor Microenvironment Profiling Identifies Prognostic Signatures and Suggests Immunotherapeutic Benefits in Neuroblastoma
Source: Front Cell Dev Biol. 2022 Apr 14;10:814836. doi: 10.3389/fcell.2022.814836 (PMC9047956; doi:10.3389/fcell.2022.814836)
Supplement: Supplementary file 2 [file DataSheet1.docx]

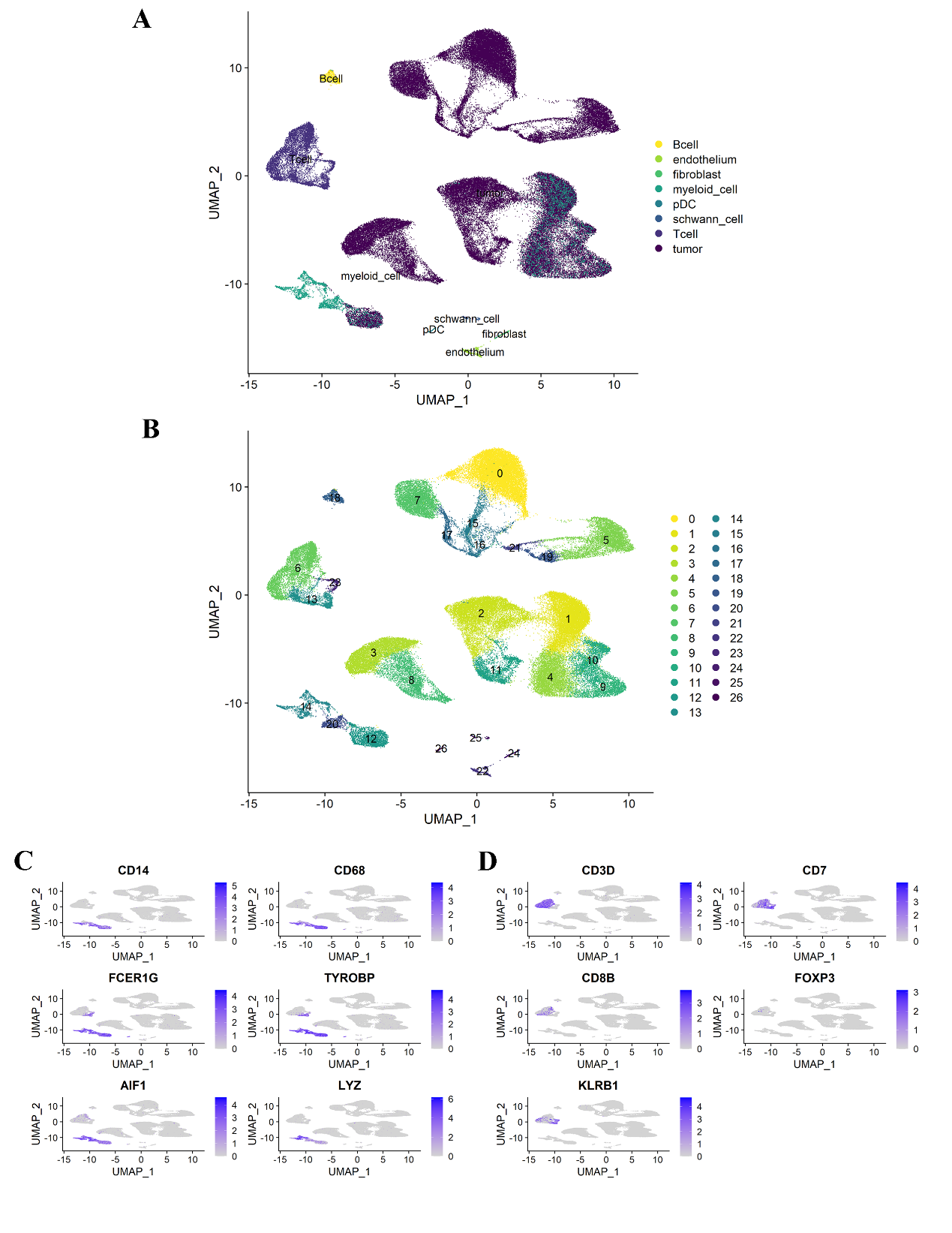


Fig.S1 Single-cell analysis depicted major cell types in the NB TME.

**A**. UMAP plot of all cells enrolled in the analysis. Cell types were annotated from GSE137804.

**B**. UMAP plot of clusters.

**C**. Expressions of selected Monocytes/Macrophages lineage markers (*CD14*, *CD68*, *FCER1G*, *TYROBP*, *AIF1*, *LYZ*) on UMAP plots.

**D**. Expressions of selected T/NK lineage markers (*CD3D*, *CD7*, *CD8B*, *FOXP3*, *KLRB1*) on UMAP plots.


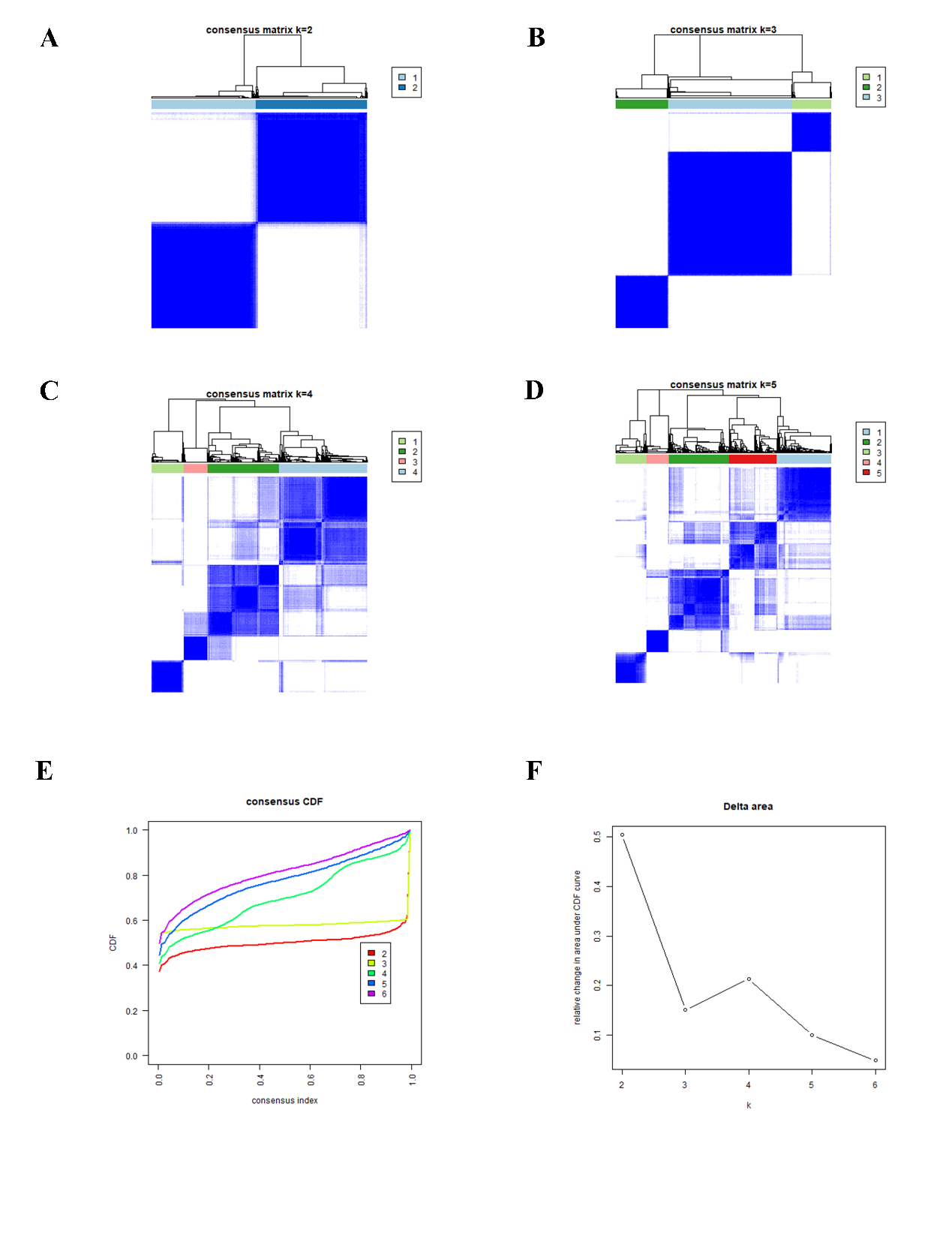


Fig.S2 Consensus clustering of TME in the SEQC cohort.

**A-D** Consensus clustering matrices for k=2-5.

**E**. The consensus CDF plot. **F**. The delta area plot.


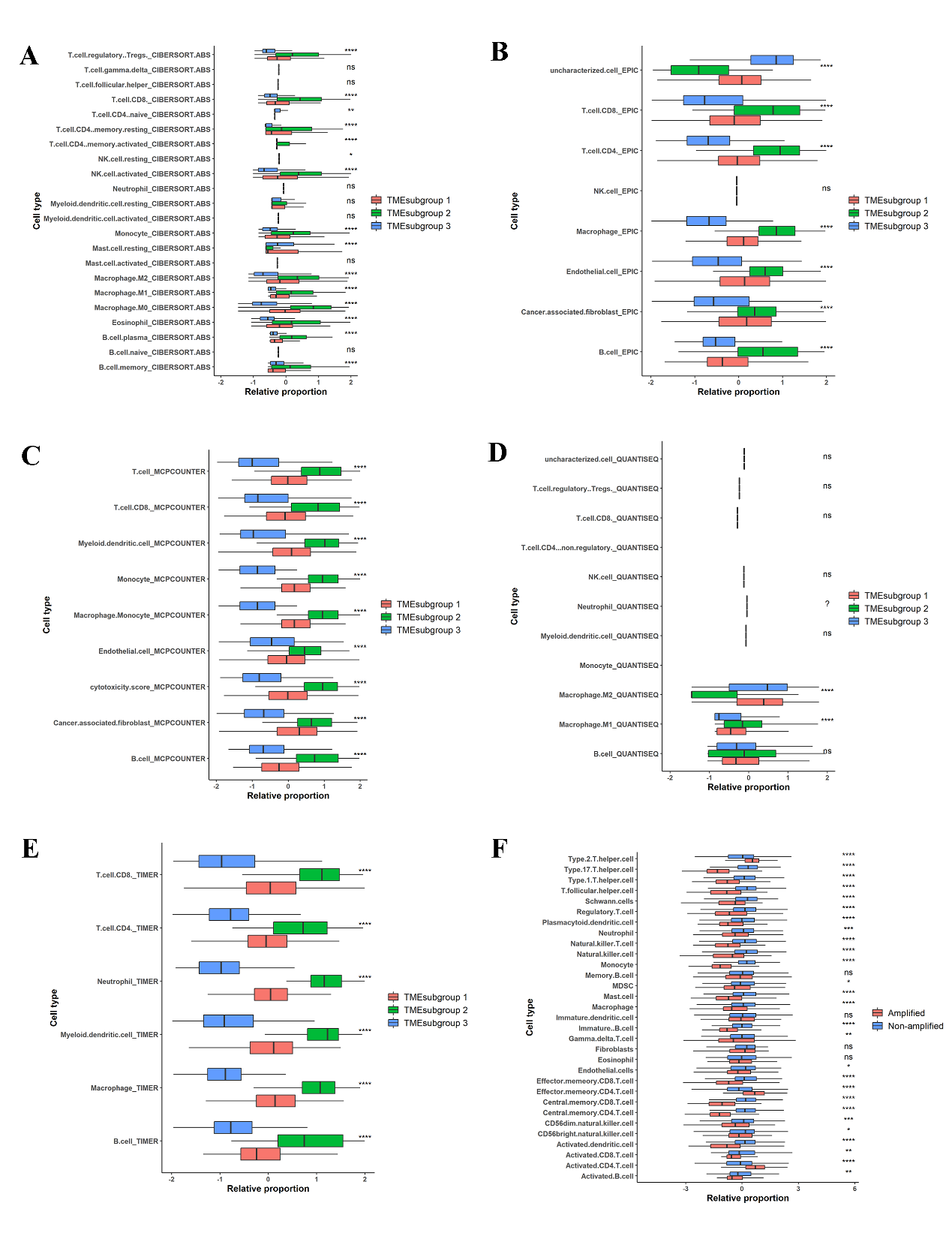


Fig.S3 Boxplots of relative cell proportions. The thick line represents the median value. The bottom and top of the boxes are the 25th and 75th percentiles. The whiskers encompass 1.5 times the interquartile range. *P*-values are labeled above each boxplot with asterisks (ns: *p* > 0.05, *: *p* < 0.05, **: *p* < 0.01, ***: *p* < 0.001, ****: *p* < 0.0001).

**A-E** Boxplots of relative cell proportion between three TMEsubgroups. Cell infiltrations were inferred by the Cibersort, EPIC, MCPCounter, QuantiSeq and TIMER algorithms, respectively.

**F**. Boxplots of relative cell proportion between *MYCN*-amplified and non-amplified groups.


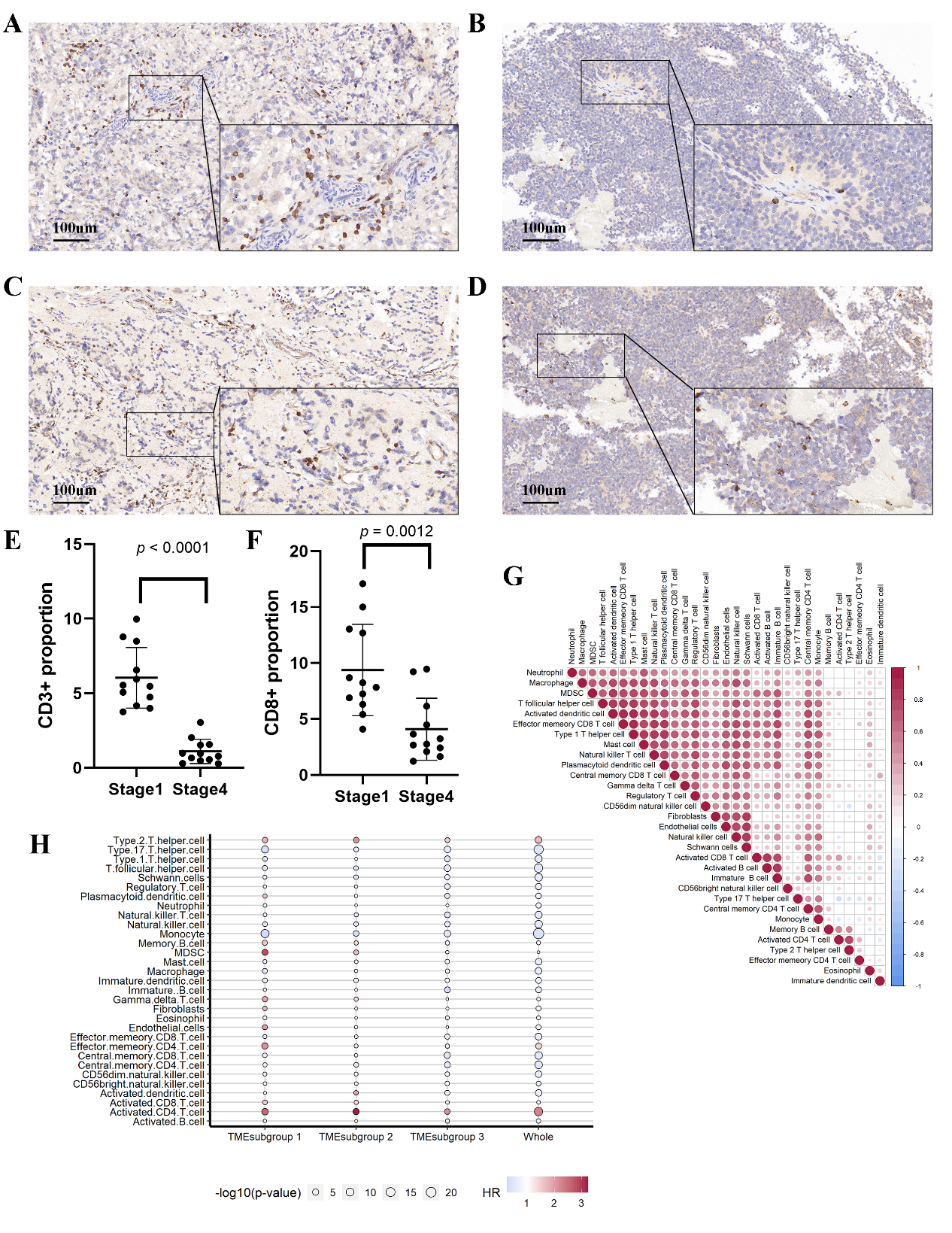


Fig.S4 Correlations and cox regression results of cell types

A-D. Representative immunohistochemistry profiling of CD3 and CD8 in stage-1 and stage-4 samples (20X). A. CD3 in stage-1 samples. B. CD3 in stage-4 samples. C. CD8 in stage-1 samples. D. CD8 in stage-4 samples. The scale bar represents 100um.

1. Heatmap of correlations between each cell type. Pearson’s correlation coefficients were used for tests.
2. Cox regression results of each cell type in 3 TMEsubgroups and whole cohort. A larger circle meant a lower p-value.


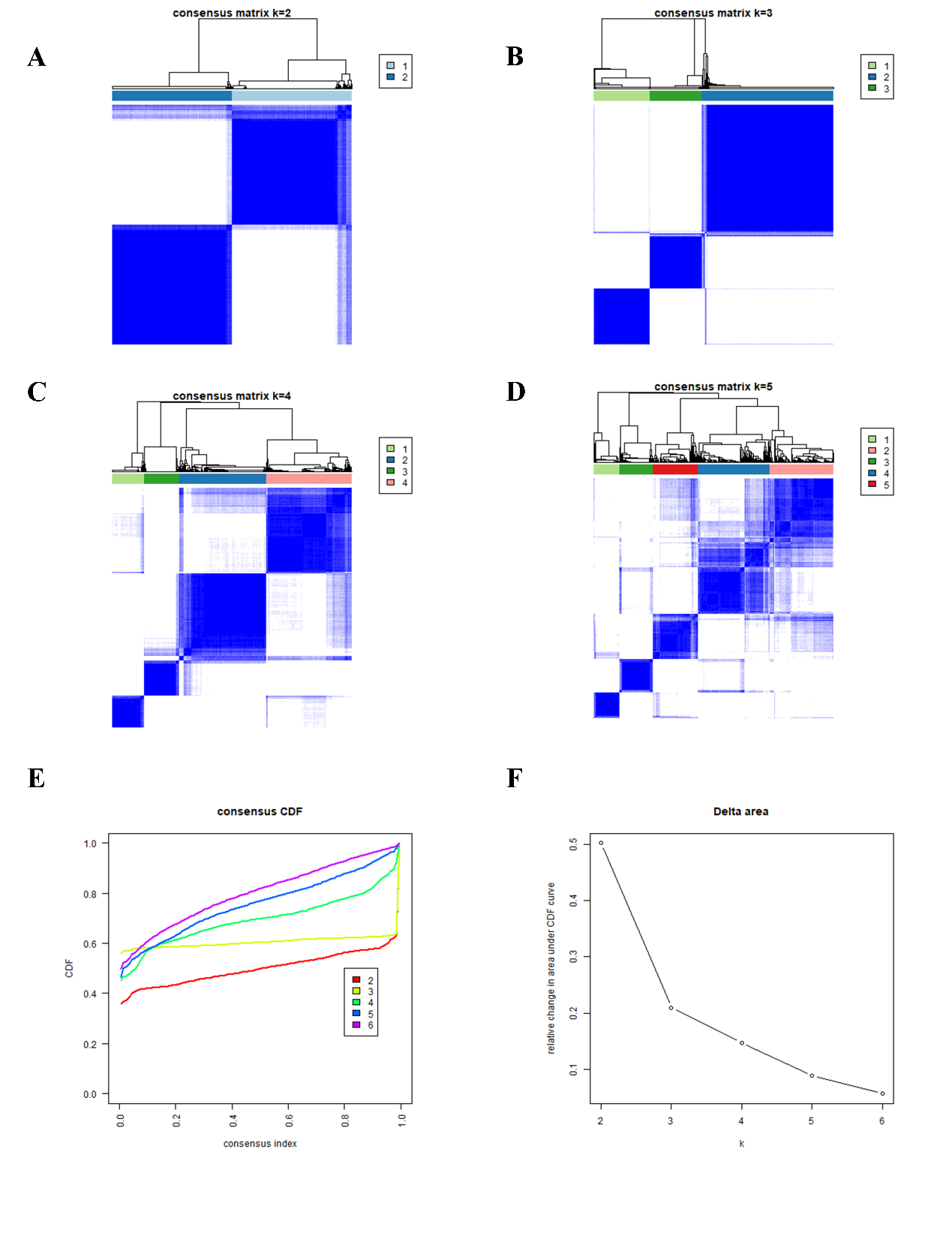


Fig.S5 Consensus clustering of DEG matrix in the SEQC cohort.

**A-D** Consensus clustering matrices for k=2-5.

**E**. The consensus CDF plot. **F**. The delta area plot.


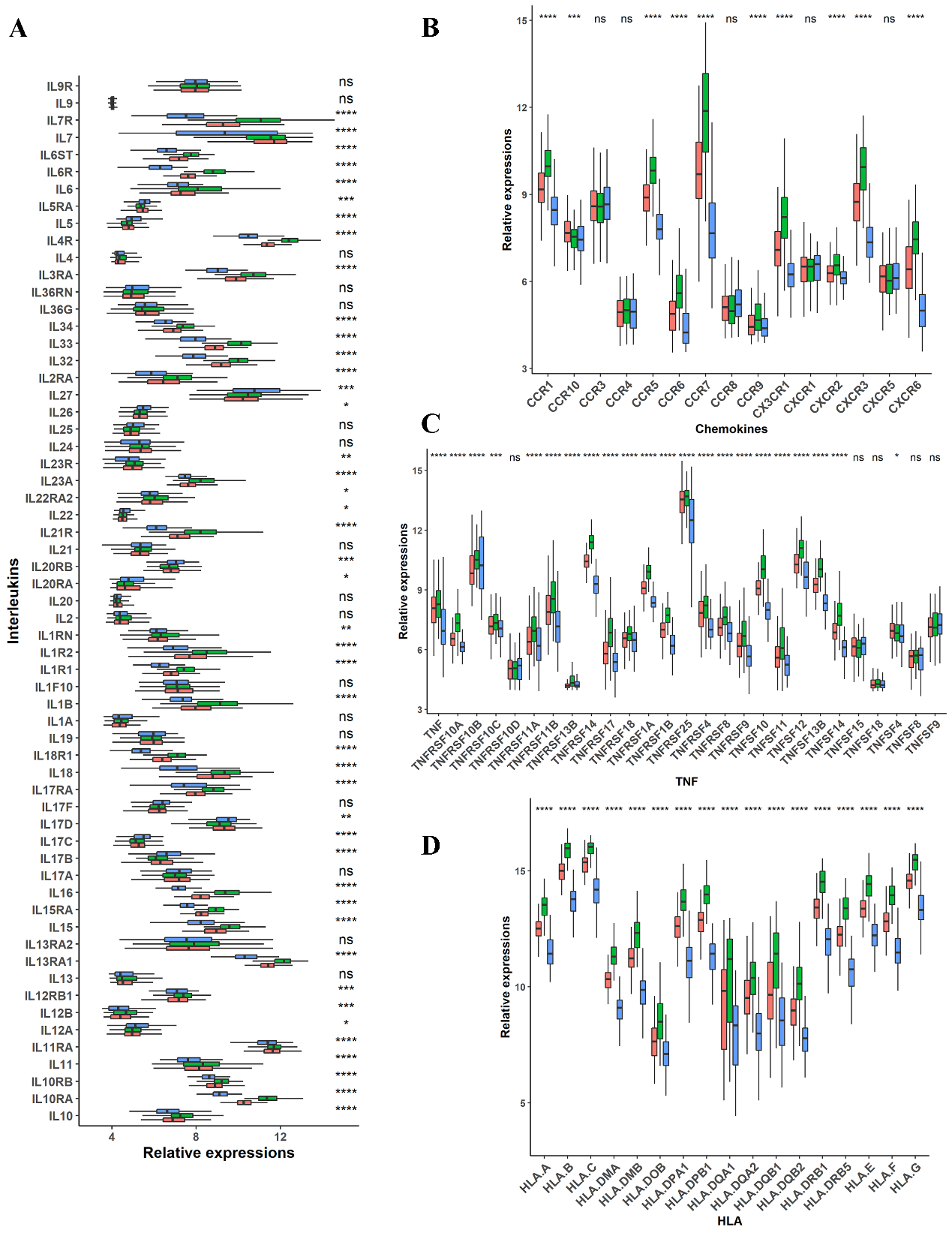


Fig.S6 Boxplots of immune-related gene expressions. The thick line represents the median value. The bottom and top of the boxes are the 25th and 75th percentiles. The whiskers encompass 1.5 times the interquartile range. *P*-values are labeled above each boxplot with asterisks (ns: *p* > 0.05, *: *p* < 0.05, **: *p* < 0.01, ***: *p* < 0.001, ****: *p* < 0.0001).

**A-D** Interleukins, chemokines, TNF super family and HLA genes, respectively.


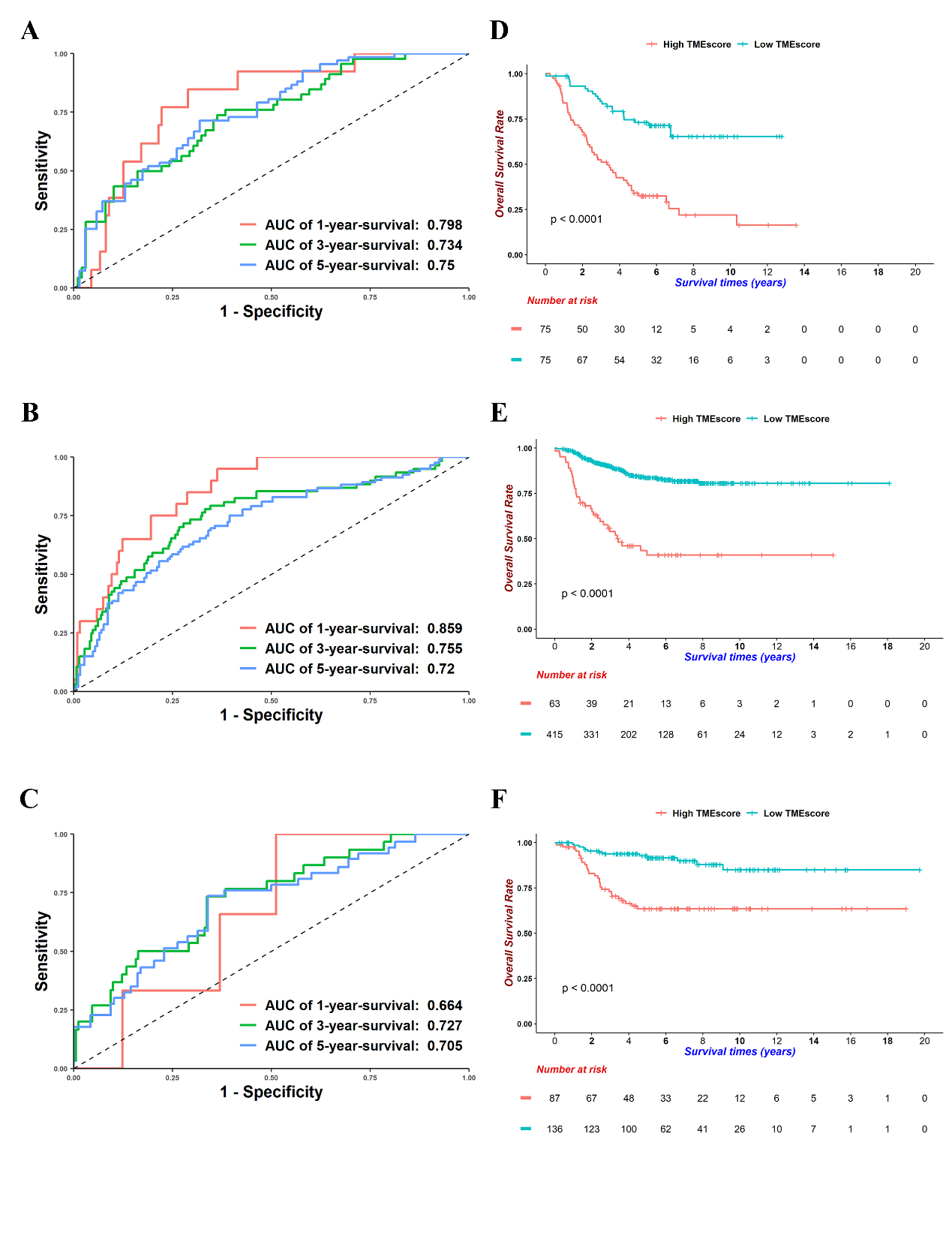


Fig.S7 ROC curves and KM curves for 3 independent NB cohorts.

1. ROC curves of TMEscore for the TARGET cohort.
2. KM curves of high- and low-TMEscore subgroups for the TARGET cohort.
3. ROC curves of TMEscore for the E-MTAB-179 cohort.
4. KM curves of high- and low-TMEscore subgroups for the E-MTAB-179 cohort.
5. ROC curves of TMEscore for the E-MTAB-8248 cohort.
6. KM curves of high- and low-TMEscore subgroups for the E-MTAB-8248 cohort.


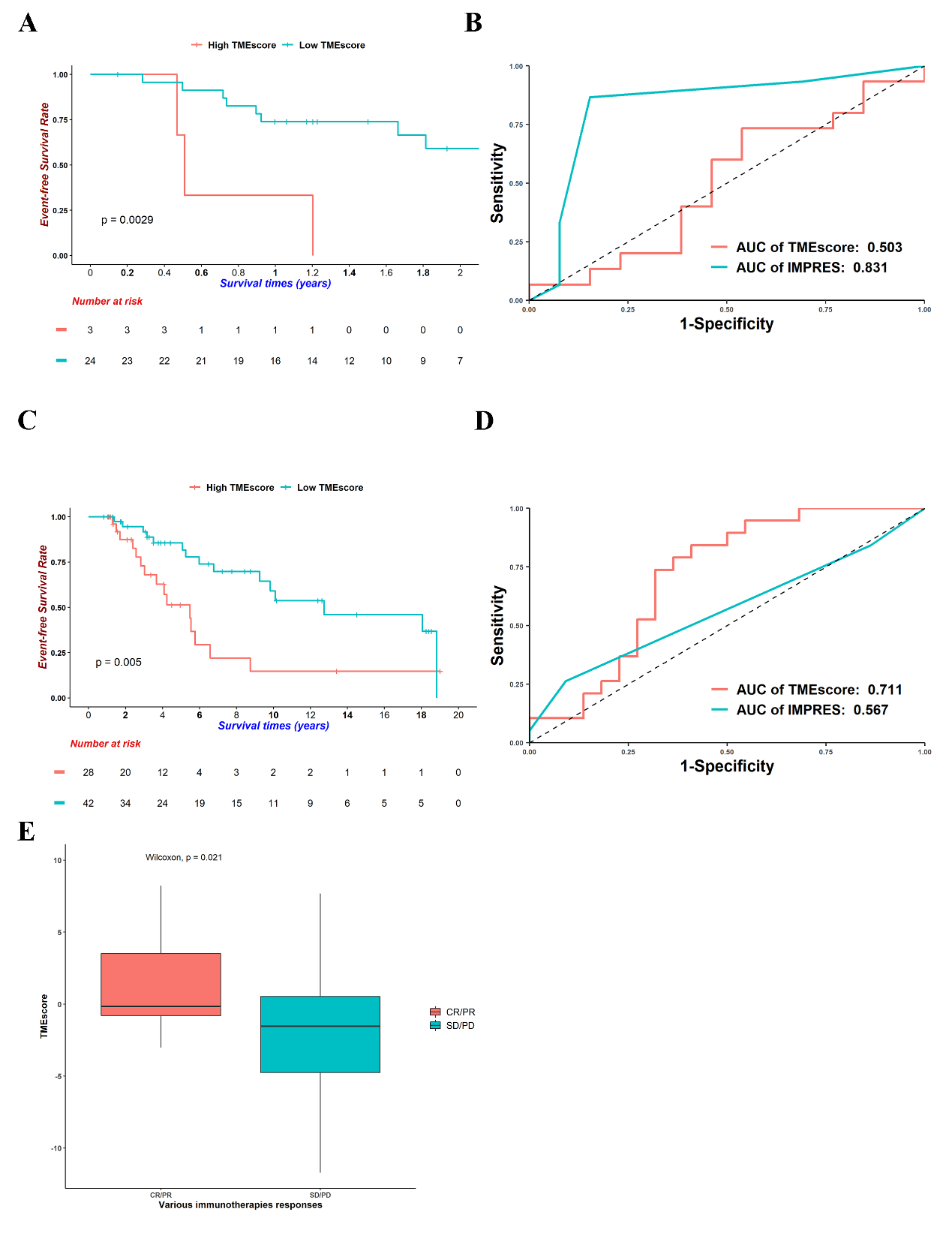


Fig.S8 The efficacy of TMEscore for predicting immunotherapy responses.

1. KM curves of high- and low-TMEscore subgroups for the anti-PD-1 cohort.
2. ROC curves of TMEscore and IMPRES for the anti-PD-1 cohort.
3. KM curves of high- and low-TMEscore subgroups for the TCGA-SKCM cohort.
4. ROC curves of TMEscore and IMPRES for the TCGA-SKCM cohort.
5. Boxplots of TMEscore between responders and non-responders in the TCGA-SKCM cohort.


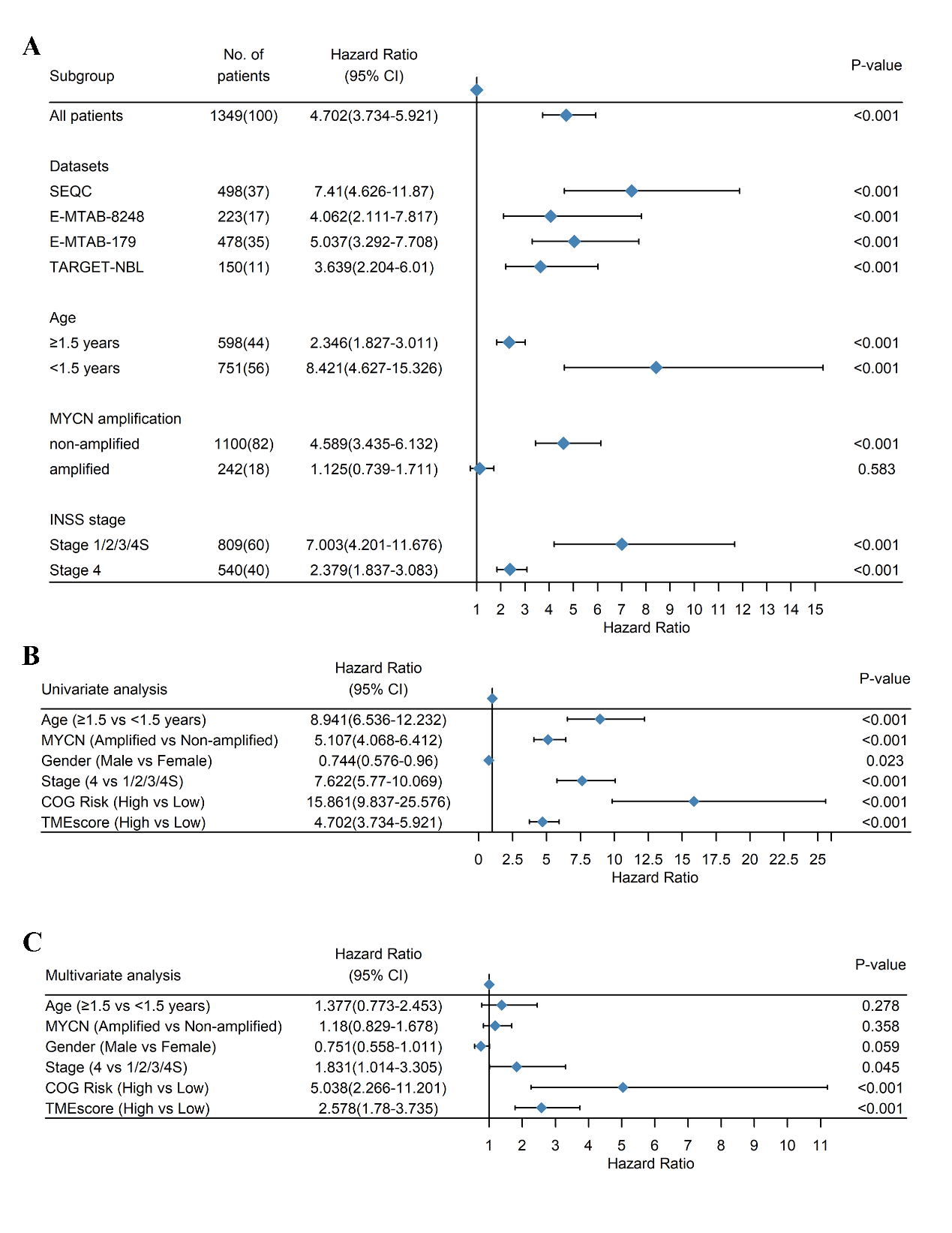


Fig.S9 Meta-analysis and clinical covariate analysis for all the NB patients.

1. Meta-analysis for the clinical covariates.
2. Univariate cox regression results of TMEscore and clinical covariates.
3. Multivariate cox regression results of TMEscore and clinical covariates.


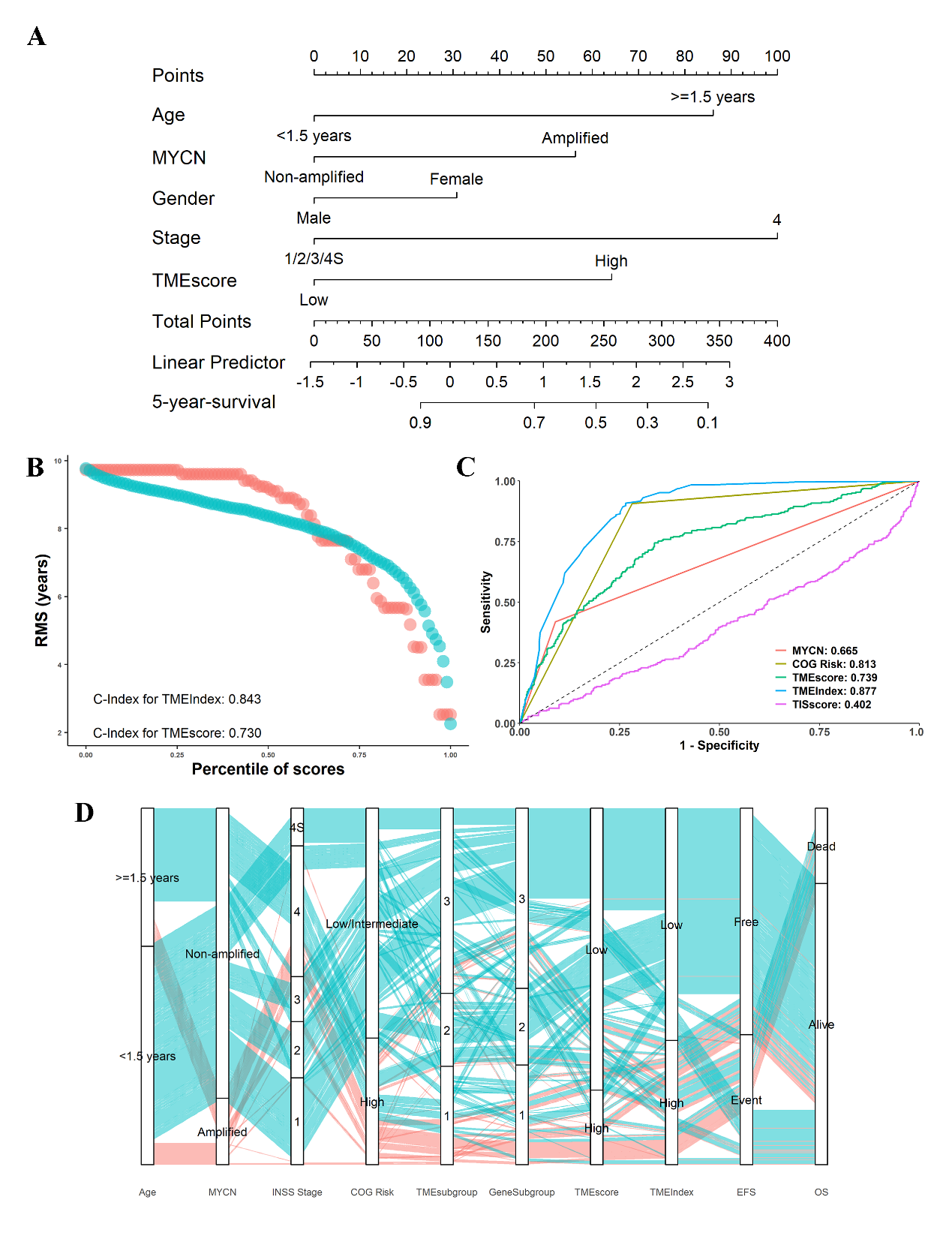


Fig.S10 Construction of TMEIndex and comparisons of signatures

1. The nomogram of 4 clinical covariates and TMEscore. The total points patients received were served as the TMEIndex.
2. RMS curves of 5-year-survivals for the TMEscore (green) and TMEIndex (red) in the SEQC patient.
3. ROC curves of MYCN, COG risk, TMEscore, TMEIndex and TIS in all NB patients.
4. Alluvial diagram of phenotypes in our analysis.
